# Non-linear Dynamical Analysis of Intraspinal Pressure Signal Predicts Outcome After Spinal Cord Injury

**DOI:** 10.3389/fneur.2018.00493

**Published:** 2018-06-26

**Authors:** Suliang Chen, Mathew J. Gallagher, Marios C. Papadopoulos, Samira Saadoun

**Affiliations:** Academic Neurosurgery Unit, Molecular and Clinical Sciences Research Institute, St. George's, University of London, London, United Kingdom

**Keywords:** chaos theory, complexity theory, critical care unit, detrended fluctuation analysis, entropy, Lyapunov, monitoring, spinal cord injury

## Abstract

The injured spinal cord is a complex system influenced by many local and systemic factors that interact over many timescales. To help guide clinical management, we developed a technique that monitors intraspinal pressure from the injury site in patients with acute, severe traumatic spinal cord injuries. Here, we hypothesize that spinal cord injury alters the complex dynamics of the intraspinal pressure signal quantified by computing hourly the detrended fluctuation exponent alpha, multiscale entropy, and maximal Lyapunov exponent lambda. 49 patients with severe traumatic spinal cord injuries were monitored within 72 h of injury for 5 days on average to produce 5,941 h of intraspinal pressure data. We computed the spinal cord perfusion pressure as mean arterial pressure minus intraspinal pressure and the vascular pressure reactivity index as the running correlation coefficient between intraspinal pressure and arterial blood pressure. Mean patient follow-up was 17 months. We show that alpha values are greater than 0.5, which indicates that the intraspinal pressure signal is fractal. As alpha increases, intraspinal pressure decreases and spinal cord perfusion pressure increases with negative correlation between the vascular pressure reactivity index vs. alpha. Thus, secondary insults to the injured cord disrupt intraspinal pressure fractality. Our analysis shows that high intraspinal pressure, low spinal cord perfusion pressure, and impaired pressure reactivity strongly correlate with reduced multi-scale entropy, supporting the notion that secondary insults to the injured cord cause de-complexification of the intraspinal pressure signal, which may render the cord less adaptable to external changes. Healthy physiological systems are characterized by edge of chaos dynamics. We found negative correlations between the percentage of hours with edge of chaos dynamics (−0.01 ≤ lambda ≤ 0.01) vs. high intraspinal pressure and vs. low spinal cord perfusion pressure; these findings suggest that secondary insults render the intraspinal pressure more regular or chaotic. In a multivariate logistic regression model, better neurological status on admission, higher intraspinal pressure multi-scale entropy and more frequent edge of chaos intraspinal pressure dynamics predict long-term functional improvement. We conclude that spinal cord injury is associated with marked changes in non-linear intraspinal pressure metrics that carry prognostic information.

## Introduction

Traumatic spinal cord injury (TSCI) is a catastrophic event that, globally, affects about 23 people per million each year (1). The management of acute TSCI is variable (2, 3) with no treatment proven to improve neurological outcome (4). To guide patient management, we developed intraspinal pressure (ISP) and spinal cord perfusion pressure (SCPP) monitoring from the injury site in the intensive care unit (ICU), analogous to intracranial pressure and cerebral perfusion pressure monitoring used to manage patients with traumatic brain injury (5, 6). The ISP monitoring technique is safe and provides clinically important information (7–16).

ISP is the pressure of the swollen, injured cord as it is compressed against the dura, whereas SCPP is the difference between mean arterial pressure (MAP) and ISP. To date we have analyzed the ISP and SCPP signals by linear techniques showing correlations between signal amplitude and injury site metabolism (11), neurological status (5), and long-term functional outcome (16). Here, we consider the injured cord from a very different angle, as a complex system influenced by many local (intraspinal compliance, cord blood flow, oxygenation, metabolism) and systemic (cardiac pulsation, aortic valve closure, respiration, plasma glucose) factors that interact over many timescales. According to chaos and complexity theories, the interactions between the different components of a complex system give rise to emergent properties that cannot be inferred from the individual parts. This idea was elegantly expressed by Aristotle in *Metaphysics* as “the whole is more than the sum of its parts.” Consider a protein with its aminoacids as the individual components. When the aminoacids are assembled in a certain sequence, the sequence folds spontaneously to form complex higher order structures that give the protein biological functions as emergent properties e.g., forming an ion channel that opens and closes in response to potential changes and ultimately gives rise to an action potential. Complex systems have unique properties, e.g., scale invariance, complexity, and self-organization, that can be quantified using concepts from chaos and complexity theories such as self-affinity (17, 18), entropy (19, 20), and stability (21, 22). There are several reviews on chaos and complexity theories for clinicians that explain these concepts in detail (23, 24).

Self-affinity determines if signal fluctuations that occur over short timescales resemble long timescale fluctuations (17, 18). We quantified self-affinity by detrended fluctuation analysis (DFA) to obtain the exponent α. If α = 0.5, ISP is uncorrelated (white noise). If α < 0.5, ISP is anti-correlated (as timescale increases, fluctuations are smaller than expected). If 0.5 < α < 1, ISP is correlated (as timescale increases fluctuations are larger than expected). If α = 1, ISP is pink noise, which normally characterizes health (25). If α > 1, ISP is non-stationary (mean amplitude changes with time). We predict that α deviates from 1 with greater cord damage.

To quantify ISP signal complexity, we computed the multiscale entropy (MSE) (19, 20). MSE yields low values for non-complex signals e.g., periodic and random ones. Based on the de-complexification theory of disease, ISP is predicted to have high complexity for healthy spinal cord and low complexity after TSCI.

Stability refers to signal predictability e.g., a sinewave is stable, white noise is chaotic. We quantified stability with the maximal Lyapunov exponent λ_max_ (21). Suppose ISP has similar values at two time-points. If the evolution of ISP from these time-points is similar, ISP is stable (λ_max_ < 0). If the evolution of ISP from these time-points diverges, ISP is chaotic (λ_max_ > 0). Health is generally associated with λ_max_~0, a special state termed edge of chaos (26, 27). At the edge of chaos, a system transitions between order and disorder. This is generally the preferred state because, unlike periodicity or randomness, edge of chaos facilitates self-organization, evolution and adaptability. Consider the brain as example of an organ that functions at the edge of chaos. The brain is orderly by using set logical rules to make deductions, but is also chaotic by producing original thoughts that may appear as if out of nowhere.

This study investigates if chaos and complexity theories can be applied to acute TSCI, if pathological events at the injury site influence non-linear ISP metrics such as MSE, α and λ_max_ and if these metrics predict functional outcome. DFA, MSE, and λ_max_ are also explained in the Supplementary Presentation.

## Materials and methods

### Approvals

Patients were part of the Injured Spinal Cord Pressure Evaluation (ISCoPE) study, which is ongoing (https://clinicaltrials.gov, NCT02721615). Approvals were obtained from the St George's, University of London Joint Research Office and the National Research Ethics Service London–St Giles Committee. The study complies with the ethical standards as laid down in the 1964 Declaration of Helsinki and its later amendments. Informed consent was obtained from all individual participants included in the study.

### Patient recruitment

All patients were treated at the Department of Neurosurgery and the Neuro-ICU at St. George's Hospital in London. ISCoPE inclusion criteria are: 1. Severe TSCI defined as American spinal injuries association Impairment Scale (AIS) grades A–C; 2. Age 18–70 years; 3. Timing between TSCI and surgery ≤ 72 h. Exclusion criteria are: 1. Unable to consent; 2. Major co-morbidities; 3 Penetrating TSCI. We included all ISCoPE patients from 2010–2016.

### Insertion of intraspinal pressure probe

**Figure 1:**
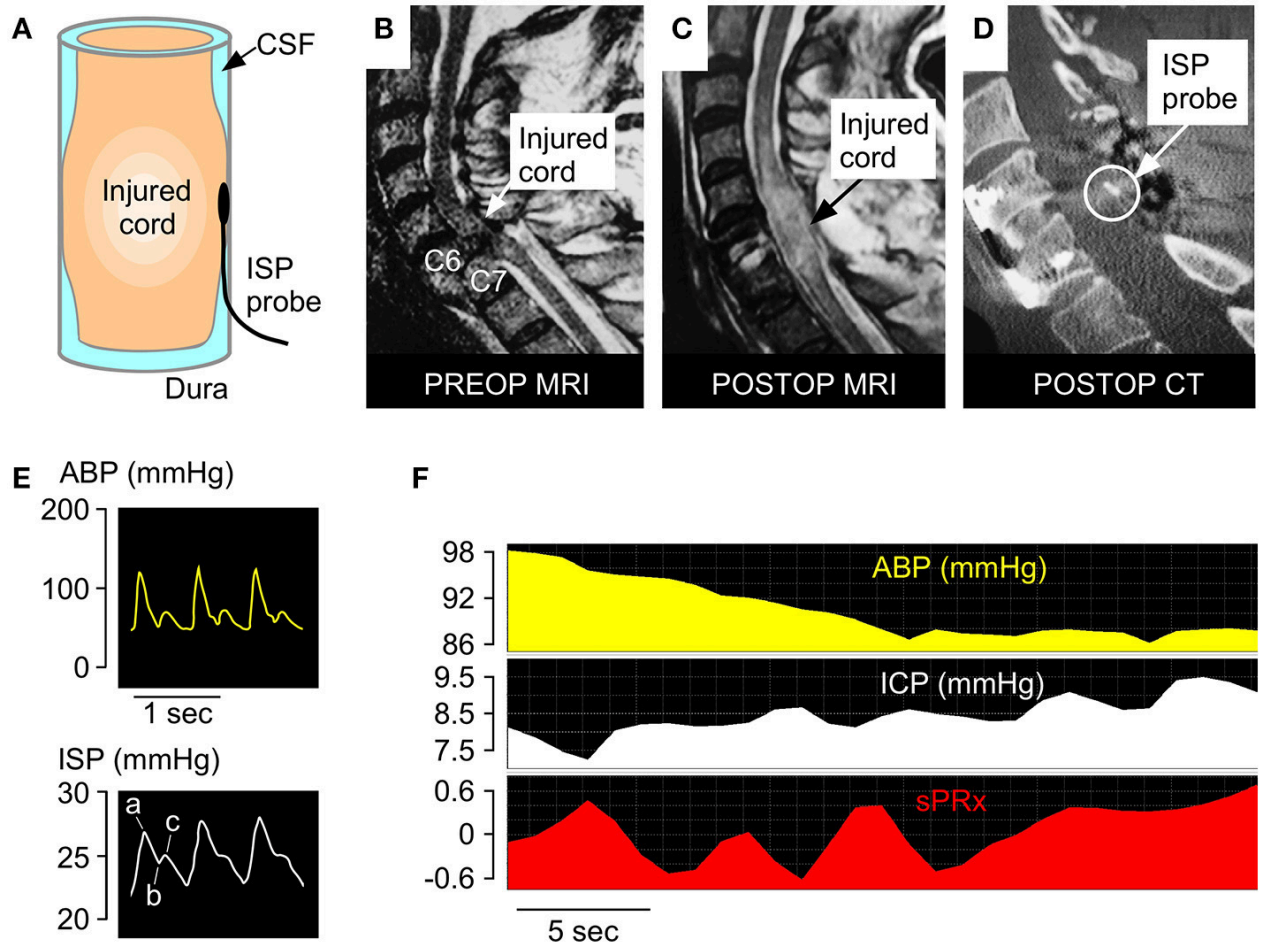
ISP monitoring technique**. (A)** Schematic showing intradurally placed ISP probe. **(B)** Pre-operative T2 MRI of a patient with TSCI at C6-7. **(C)** Postoperative T2 MRI of same patient. **(D)** Postoperative CT of same patient with ISP probe *in situ*. **(E)** Examples of ABP (yellow) and ISP (white) signals. Percussion peak (a), dicrotic notch (b), dicrotic peak (c). **(F)** Examples of ABP, ISP, and sPRx recordings.

After bony realignment and posterior fixation, the ISP probe (Codman Microsensor Transducer®, Depuy Synthes, Leeds, UK) was placed intradurally on the spinal cord surface at the site of maximal cord swelling. The probe monitors pressure, which is generated by the swollen injured cord compressed against the dura. These ISP recordings differ from corresponding values obtained from proximal or distal cord or extradurally. The ISP monitoring technique is illustrated in Figure 1. Details are given elsewhere (5, 6, 9–12, 15, 16).

### Intraspinal pressure, spinal cord perfusion pressure

The ISP probe was connected to a Codman ICP box linked via a ML221 amplifier to a PowerLab running LabChart v.7.3.5 (AD Instruments, Oxford, UK). Arterial blood pressure was recorded from a radial artery catheter kept at the same level at the ISP probe and connected to a Philips Intellivue MX800 bedside monitoring system (Philips, Guildford, UK) in turn connected to the PowerLab system. ISP and arterial blood pressure signals were sampled at 1 kHz for up to 7 days. LabChart was used to analyse the signals and compute SCPP as mean arterial pressure (MAP) minus ISP. Spinal pressure reactivity index (sPRx) was computed as the running correlation coefficient over 5 min between MAP and ISP. Details are given elsewhere (5, 6, 9–12, 15, 16).

### Patient assessments

Neurological examinations to record the AIS grade were done on admission, prior to discharge to the spinal rehabilitation center and during outpatient appointments. A CT and MRI of the whole spine were done on admission, another CT within 48 h of surgery and another MRI within 2 weeks of surgery.

### Detrended fluctuation analysis

We used DFA to compute the scaling exponent α (17, 18). A program for conducting DFA was created in Matlab. Each ISP signal was divided into non-overlapping 1 h windows, i.e., *N* = 3,600 at 1 Hz sampling rate. Each 1 h window, the ISP was normalized using z scores and integrated by computing the cumulative sum to produce the time series *X(t)*. The normalized and integrated time series *X(t)* was then divided into time segments of equal length *n* and a least square linear best fit was performed in each time segment to obtain the local trend *Y(t)*. The normalized and integrated time series *X(t)* was detrended by subtracting the local trend *Y(t)* in each time segment. The fluctuation *F(n)* defined as the root-mean-square deviation from the mean was computed as follows:

(1) $$F\left(n\right)=\sqrt{\frac{1}{N}\sum_{t=1}^{N}{\left(X\left(t\right)-Y\left(t\right)\right)}^{2}}$$

Detrending followed by fluctuation measurement was repeated for a range of segment sizes n from 100 to 1,000 with interval 100 and a log[*F*(*n*)] vs. log (*n*) plot was constructed. The exponent α was calculated as the slope of the linear regression fitted to the log [*F*(*n*)] vs. log (*n*) plot. DFA was performed in each 1-h time window to obtain α.

### Multi-scale entropy

MSE measures the complexity of a time series (19, 20). To calculate MSE, we used the software of ICM+ (www.neurosurg.cam.ac.uk/icmplus). MSE analysis starts with a coarse-graining process applied to the time series. For each 1 h ISP time series, the ISP signal sampled at 1 Hz was normalized using z scores. Multiple coarse-grained time series were then generated by averaging the data points within non-overlapping windows of increasing length. Each 1 h ISP time series was investigated with scale factors 1–20. Sample entropy was computed for each coarse-grained time series with non-overlapping windows. For a given coarse-gained time series, sample entropy takes into account the number of repeated m (embedding dimension) consecutive data points (m = 2 in the study), given by A, and the number of repeated m+1 consecutive points, given by B. Sample entropy, defined as $-\log_{e}\left(\frac{A}{B}\right)$, is a regularity measure that searches for similar patterns. Sample entropy was computed for each scale and plotted against scale factor. A single value, termed MSE, was computed as the area under the sample entropy vs. scale factor curve.

### Maximum lyapunov exponent

The maximum Lyapunov exponent λ_max_ is an important statistic for quantifying stability and distinguishing chaotic from non-chaotic behavior in dynamical systems (21, 22). Here, we consider the ISP signal to be stable when λ_max_ < −0.01, chaotic when λ_max_ > 0.01 and at the edge of chaos when −0.01 ≤ λ_max_ ≤ 0.01. A program for calculating ISP λ_max_ was created in Matlab based on the computation process of Wolf et al. (21) that does not require fitting a model to the time series. The ISP signal, sampled at 0.1 Hz and divided into 1-h non-overlapping windows, was normalized using z-scores. The parameters chosen for the algorithm are embedding dimension m = 3 and time delay τ = 10 s. Time delay reconstruction defines the tuples x(i), x(i+τ), x(i+2τ) etc. from the time series x(i) and the reconstructed phase space consists of points in m-dimensional space. The value of m should not be too small that causes the reconstruction to be topologically incorrect and was chosen has the 3D space in current study.

### Statistical analysis

We used XLStat Biomed (v.18.07, Addinsoft, New York). Regression lines were fitted and Pearson's correlation coefficient was computed. Logistic regression was performed using Logit with binary response (AIS improvement / no improvement) to fit the best model based on the likelihood ratio.

### Data availability

The datasets generated during and/or analyzed during the current study are available from the corresponding author on reasonable request.

## Results

### Patient characteristics

We analyzed 5,941 h of ISP data obtained from 49 TSCI patients. Most patients were young men with complete, i.e., AIS grade A, cervical TSCI on admission. All patients underwent posterior surgery to fix the spine, most within 48 h of the injury, and were followed up for about one and a half years on average. ISP was monitored for about 5 days on average. Overall, at follow-up 19/49 (38.8%) patients had improved by at least one AIS grade, including (8/34) 23.5% who were AIS grade A, 4/7 (57.1%) who were AIS grade B and 7/8 (87.5%) who were AIS grade C on admission. For details see Table 1.

**Table 1:** Patient characteristics.

**Characteristic**	**Value**	**Percent**
No. of patients	49	
Age in years: mean ± sem	41.1 ± 2.1	
Sex: male, female	39, 10	79.6, 20.4
Level: cervical, thoracic	27, 22	55.1, 44.9
Surgery: posterior, anterior, both	41, 0, 8	83.7, 0, 16.3
Injury to surgery: hours mean ± sem	36.4 ± 2.7	
Monitoring: hours mean ± sem	121.2 ± 5.9	
Admission AIS grade: A, B, C	34, 7, 8	67.3, 14.3, 18.4
Follow-up AIS grade: A, B, C, D, E	26, 4, 6, 12, 1	53.1, 8.2, 12.2, 24.5, 2.0
Months follow-up: mean ± sem	17.2 ± 2.3	

### Detrended fluctuation analysis

**Figure 2:**
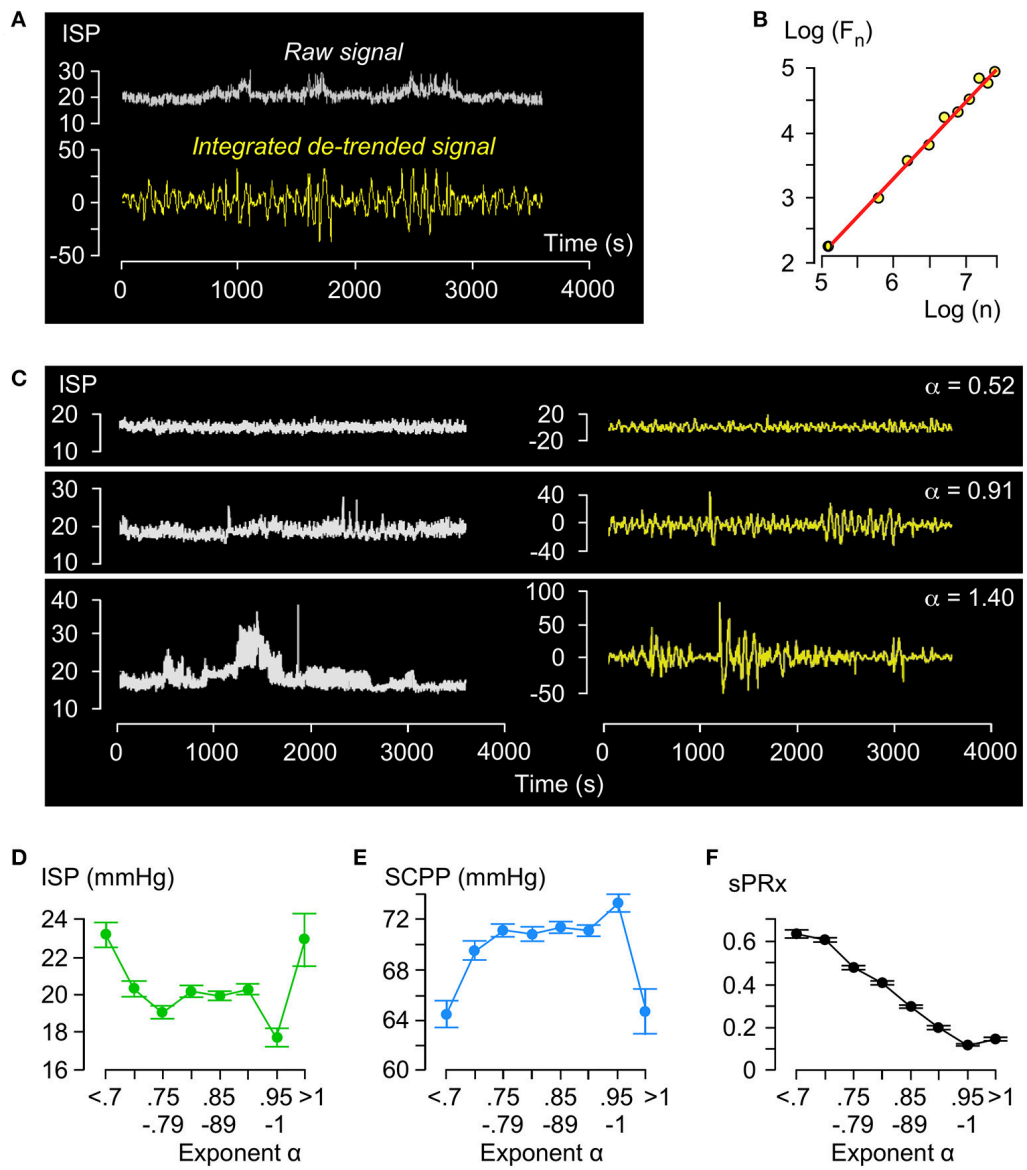
Detrended fluctuation analysis of ISP signal**. (A)** Example of raw (white, top) and integrated detrended (yellow, bottom) ISP signal. **(B)** Log F(n) vs. Log n plot and trend-line (*R* = 1.00, slope = α). **(C)** Raw (white, left) and integrated detrended (yellow, right) ISP signals with increasing α. **(D)** ISP vs. α (green). Trend-line *R* = −0.18. **(E)** SCPP vs. α (blue). Trend-line *R* = 0.19. **(F)** sPRx vs. α (black). Trend-line *R* = −0.98. For **(C–E)** *n* = 49 patients, mean±standard error.

Figure 2A illustrates the principles of DFA. The raw ISP signal is integrated and detrended and the root-mean-square fluctuation F_n_ computed by partitioning the processed curve into n non-overlapping time windows. By plotting Log (F_n_) vs. Log(n), we obtain a trend-line with gradient α (Figure 2B). Trend-lines fit the Log (F_n_) vs. Log(n) points well (*R* > 0.9) and values of α ranged between 0.55 and 1.40 with mean of 0.85. Figure 2C shows examples of ISP signals with different α. Fewer than 2% of α values were >1. Thus, regarding the amplitude fluctuations in the ISP signal: (1) Most (98%) of the time there is long-term positive temporal correlation, i.e., 0.5 < α < 1; (2) There is never long-term negative correlation, i.e., α is never < 0.5; (3) Non-stationarity, i.e., α > 1.0, which means that the mean and variance of the amplitude vary with time occurs rarely (< 2% of the time). Non-stationary hours cluster in a few patients (87% in 6 patients) and are not pathological because all patients with mean α > 1.0 improved by at least 1 AIS grade.

Relationships between ISP vs. α (Figure 2D) and SCPP vs. α (Figure 2E) are non-linear: as α increases from 0.5 to 0.8, ISP decreases and SCPP increases, but as α increases from 0.8 to 1, ISP and SCPP plateau. With α > 1, ISP increases and SCPP decreases. There is near-perfect negative correlation between sPRx vs. α (Figure 2F). Therefore, as the injured cord swells or becomes ischaemic or the vascular pressure reactivity is impaired, the long-term correlations in amplitude dynamics of the ISP signal become attenuated. α>1 is a unique state of low sPRx despite high ISP and low SCPP.

### Complexity analysis

**Figure 3:**
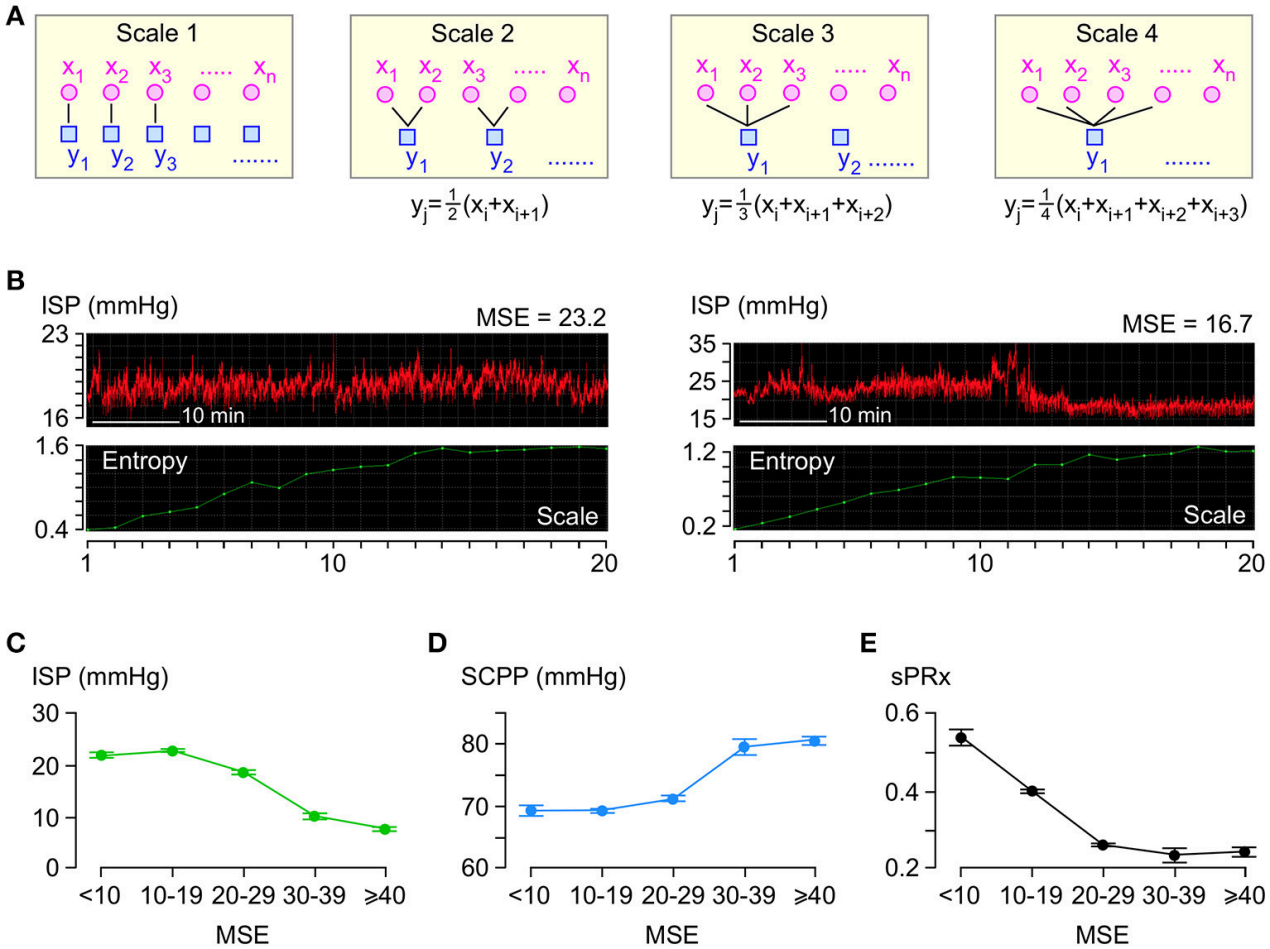
Entropy analysis of ISP signal. **(A)** Coarse-graining process: For each 1 h long ISP signal, multiple coarse-grained time series are generated by averaging data points *x*_*i*_ within non-overlapping windows of increasing time length to obtain *y*_*j*_. **(B)** Examples of two raw ISP signals (red, top) and their corresponding sample entropy (MSE) vs. scale plots (green, bottom). **(C)** ISP vs. MSE (green). Trend-line *R* = −0.94. **(D)** SCPP vs. MSE (blue). Trend-line *R* = 0.93. **(E)** sPRx vs. MSE (black). Trend-line *R* = −0.90. For **(C–E)** *n* = 49 patients, mean±standard error.

Figure 3A illustrates the coarse-graining process. For each 1 h long ISP signal, several coarse-grained time series are generated by averaging the data points within non-overlapping time windows of decreasing length as follows: scale factor 1 means 1 window which is 1 h long, scale factor 2 means 2 windows which are $\frac{1}{2}$ h long each, scale factor 3 means 3 windows which are $\frac{1}{3}$ h long each etc. We plot the sample entropy of each coarse-grained time series vs. the scale factor and compute MSE as the area under the curve. Examples of ISP signals with low and high MSE are in Figure 3B. Though the MSE of the ISP signals varies widely (4.1–298.7), most (95.5%) MSE values are <30.

Though the relationships between ISP vs. MSE, SCPP vs. MSE, and sPRx vs. MSE are non-linear (Figures 3C–E), there is significant negative correlation between ISP vs. MSE and between sPRx vs. MSE and significant positive correlation between SCPP vs. MSE. Therefore, as the injured cord swells or becomes more ischaemic or as the pressure reactivity becomes more impaired, the ISP signal becomes less complex, i.e., the number of interacting biological processes that influence ISP decreases.

### Stability analysis

**Figure 4:**
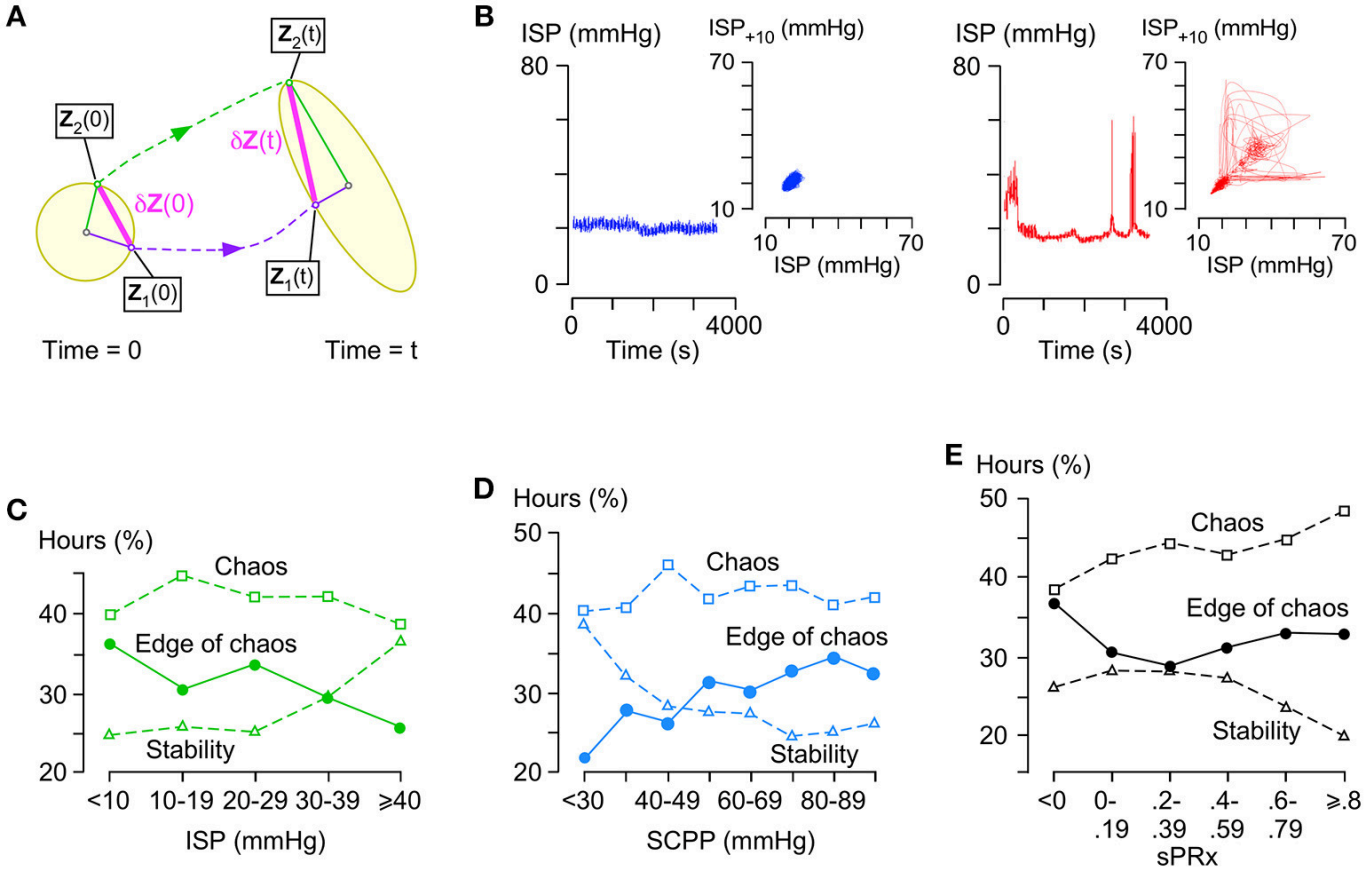
Stability analysis of ISP signal. **(A)** Concept of Lyapunov exponents: In phase space, a small n-dimensional sphere with radius *p*_1_(0) = *p*_2_(0) = … = *p*_*n*_(0) at time 0 becomes an ellipsoid with radii *p*_1_(*t*), *p*_2_(*t*), …, *p*_*n*_(*t*) at time t. The i-th Lyapunov exponent is defined as $\lambda_{i}=\underset{t\to \infty }{\lim}\left(\frac{1}{t}\right)\log\frac{p_{i}\left(t\right)}{p_{i}\left(0\right)}$. **(B)** Examples of stable (left, blue, λ_max_ < -0.01) and chaotic (right, red, λ = 0.06) ISP signals with their respective phase space trajectories (insets, 10 s time delay embedding). **(C)** % hours which are at edge of chaos (circles, −0.01 ≤ λ_max_ ≤ 0.01), stable (triangles, λ_max_ < −0.01), or chaotic (squares, λ_max_ > 0.01) vs. ISP. Trend-line *R* = −0.86 (edge of chaos), 0.87 (stability), −0.36 (chaos). **(D)** % hours which are at edge of chaos (circles, −0.01 ≤ λ_max_ ≤ 0.01), stable (triangles, λ_max_ < −0.01), or chaotic (squares, λ_max_ > 0.01) vs. SCPP. Trend-line *R* = 0.89 (edge of chaos), −0.84 (stability), 0.10 (chaos). **(E)** % hours which are at edge of chaos (circles, −0.01 ≤ λ_max_ ≤ 0.01), stable (triangles, λ_max_ < −0.01), or chaotic (squares, λ > 0.01) vs. sPRx. Trend-line *R* = 0.19 (edge of chaos), −0.76 (stability), 0.91 (chaos). For **(C–E)** 49 patients.

Figure 4A illustrates the concept of λ_max_ in phase space, which is an N-dimensional plot of all ISP trajectories. Consider two states in the phase space at time 0 located at **Z**_1_(0) and **Z**_2_(0) with a very small separation δ**Z**(0). After time t, the two states are located at **Z**_1_(t) and **Z**_2_(t), respectively, with separation δ**Z**(t).

(2) $$\left|\delta Z(\text{t})\right|=e^{\lambda t}\left|\delta Z(0)\right|$$

λ is a Lyapunov exponent and the number of λ values is N. The largest λ is given by

(3) $$\lambda_{\max}=\underset{t\to \infty }{\lim}\underset{\delta Z(0)\to 0}{\lim}\frac{1}{\text{t}}\ln\frac{\left|\delta Z(t)\right|}{\left|\delta Z(0)\right|}$$

and determines if the system is chaotic. Figure 4B shows two ISP signals with their corresponding trajectories in phase space as seen in two dimensions; one ISP signal is stable (λ_max_ < 0) and the other chaotic (λ_max_ > 0).

λ_max_~0 indicates a special state, termed edge of chaos, which is preferred in healthy biological systems. Here, we defined edge of chaos as −0.01 ≤ λ_max_ ≤ 0.01. We found significant negative correlation between the % hours at the edge of chaos vs. ISP; as ISP increases, the ISP signal is less frequently at the edge of chaos and more frequently stable (Figure 4C). There was significant positive correlation between the % hours at the edge of chaos vs. SCPP; as SCPP increases, the ISP signal is more frequently at the edge of chaos and less frequently stable (Figure 4D). Though there was no correlation between the % hours at the edge of chaos vs. sPRx; however, as sPRx increases, the ISP signal becomes less stable and more chaotic (Figure 4E). These findings suggest that as the injured cord swells or becomes ischaemic, ISP loses its edge of chaos dynamics by becoming more regular. As the vascular pressure reactivity at the injury site becomes impaired, ISP is less regular and more chaotic.

### Patient prognosis

**Figure 5:**
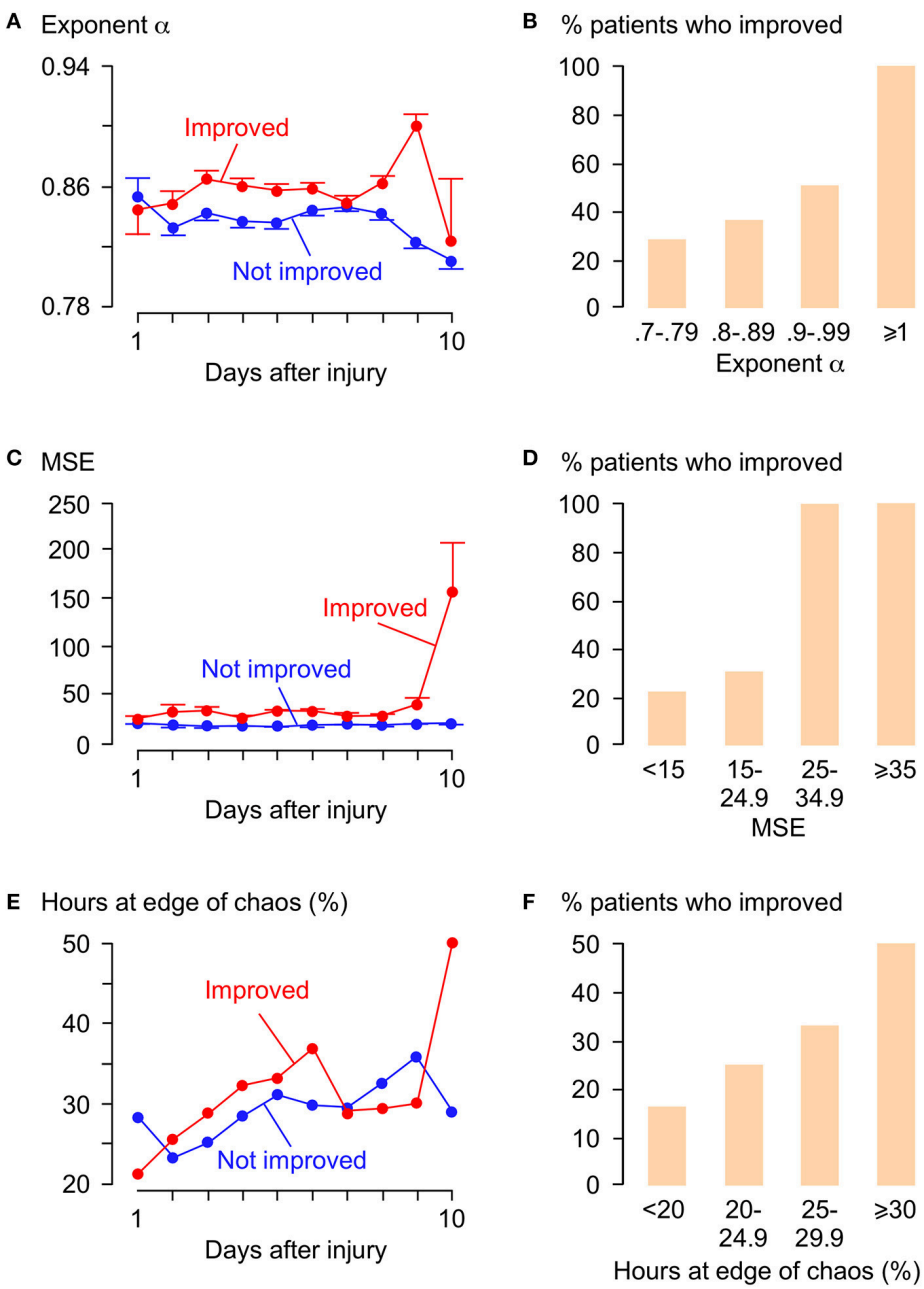
Relations between α, MSE, λ_max_, and functional outcome. **(A)** α vs. days after TSCI. **(B)** % patients who improved vs. α. Trend-line *R* = 0.92. **(C)** MSE vs. days after TSCI. **(D)** % patients who improved vs. MSE. Trend-line *R* = 0.92. **(E)** λ vs. days after TSCI. **(F)** % patients who improved vs. % hours at edge of chaos (−0.01 < λ_max_ < 0.01) . Trend-line *R* = 0.98. For **(A,C,E)** Patients who improved by ≥1 AIS grade (red, 19 patients, 1,996 h) and patients not improved (blue, 30 patients, 4,068 h), mean±standard error.

Figure 5 shows plots of α, MSE and % hours at the edge of chaos vs. time for those patients who improved by at least 1 AIS grade and those who did not. Overall, patients who improved have significantly larger α, significantly larger MSE and significantly higher % hours at the edge of chaos than patients who did not improve. Interestingly, large rises in MSE (~3.0-fold) and % hours at the edge of chaos (~1.7-fold) are seen at day 10 in patients who improve, but not in patients who do not improve. The significance of this is unclear; it may indicate some important physiological change at the injury site or may be an artifact due to the small number (= 11) of data points at this time point. There are also positive correlations between % patients who improved vs. mean α, vs. mean MSE, and vs. mean % hours at the edge of chaos. Logistic regression analysis is summarized in Table 2. Univariate regression identified patient age, admission AIS grade, mean ISP, mean SCPP, mean exponent α, mean MSE, and overall % hours at the edge of chaos as prognostic factors after TSCI. According to a multivariate regression, admission AIS grade, mean MSE, and overall % hours at the edge of chaos are significant independent prognostic factors.

**Table 2:** Univariate and multivariate logistic regression analysis.

**Variable**	**Univariate**		**Multivariate**	
	***P*-value**	**Odds ratio**	***P*-value**	**Odds ratio**
Age	<0.01	0.49 (0.27–0.84)	NS	
Admission AIS	<0.0005	4.65 (1.91–14.55)	<0.005	6.73 (1.81–51.50)
ISP	<0.05	0.26 (0.09–0.61)	NS	
SCPP	<0.05	2.21 (1.19–4.49)	NS	
Exponent α	<0.05	2.71 (1.13–8.01)	NS	
MSE	<0.0001	6.54 (1.26–38.77)	<0.0005	6.10 (1.96–39.61)
Hours−0.01 < λ_max_ < 0.01	<0.05	2.62 (1.05–8.68)	<0.05	4.94 (1.30–34.25)

*Exponent α, detrended fluctuation analysis exponent; ISP, mean hourly intraspinal pressure; λ_max_, maximum Lyapunov exponent; MSE, multi scale entropy; NS, not significant; SCPP, mean hourly spinal cord perfusion pressure*.

## Discussion

Our key findings are that pathological changes occurring after TSCI at the injury site (cord swelling, ischaemia, impaired spinal pressure reactivity) produce marked changes in non-linear ISP metrics including α, MSE and λ_max_, and these metrics have prognostic value.

Central to our study is the concept of the spinal cord as a complex system with several components that interact over timescales ranging from seconds to hours to produce the ISP time series. Studies of TSCI normally reduce the spinal cord to its individual parts, i.e., cells or molecules, study their behavior, and re-assemble them to form the whole. At the macroscopic level, complex systems have emergent properties that are absent from the individual components, hence “the whole is more than the sum of its parts.” The emergent properties may also inhibit the component parts and thus “the whole is also less than the sum of its parts.” These ideas suggest that the response of ISP to TSCI cannot be fully understood by studying the individual components of the spinal cord in isolation. Chaos and complexity theories provide a novel perspective on TSCI with a set of conceptual tools to tackle the problem. One such concept is self-similarity, i.e., the system exhibits the same behavior at different timescales, the hallmark of which is the power law (17, 18). We showed that the ISP signal is self-similar by obeying power laws with α > 0.5. We hypothesized that the ISP signal carries information over different timescales that arises from the several biological processes that influence it. We thus quantified the information content of the ISP signal over several scales using MSE (19, 20). Another concept in complex systems is edge of chaos dynamics (21, 22), i.e., the spinal cord fluctuates at the interface between order and disorder. This state, also known as self-organized criticality, is an emergent property and is the preferred state of healthy biological systems because it accelerates information processing and storage (27) as well as maximizing adaptability to changing demands (26). We showed that, for long periods, the ISP signal is at the edge of chaos with λ_max_~0. Together, our data suggest that the spinal cord is a complex system that generates an ISP signal with high self-affinity, high information content (high MSE) and edge of chaos dynamics.

We found that secondary insults disrupt the self-affinity, the complexity and the edge of chaos dynamics of the ISP signal. The secondary insults studied here are cord compression (increased ISP), ischaemia (decreased SCPP) and impaired autoregulation (increased sPRx). These insults likely disrupt the fundamental biological processes that regulate the ISP signal, which in turn cause the observed changes in α, MSE and λ_max_. Consequently, as these insults become more severe, the self-affinity of ISP weakens (α decreases), the ISP signal loses information (MSE decreases) and becomes more regular or chaotic (fewer hours with −0.01 ≤ λ_max_ ≤ 0.01). We showed that α, MSE and λ_max_ carry prognostic information, probably because these metrics are sensitive to the pathological processes at the injury site. The multivariate logistic regression model shows that the chance of AIS grade conversion at follow-up is increased 6.7-fold when AIS grade at presentation increases by one, when MSE increases by 10 or when the number of hours with −0.01 ≤ λ_max_ ≤ 0.01 increases by 5%.

Chaos theory has not been previously applied to TSCI because ISP monitoring is new. The idea of applying chaos theory to traumatic brain injury has been proposed in reviews (28), but few such studies have been undertaken. Reduced MSE (29) or α >1 (30, 31) in the ICP signal is associated with worse Glasgow Outcome Score after traumatic brain injury. Decreased MSE of near infrared spectroscopy signals obtained from the brains of critically ill preterm infants correlates with death and brain damage (32). Together, these findings suggest that non-linear ISP metrics are clinically useful in acute traumatic brain injury as well as TSCI.

Some issues need to be addressed before non-linear metrics can be used clinically. Foremost is the large number of computations required that may preclude non-linear analysis to be done in real time. Another practical issue is that a change in ISP complexity, which signifies an adverse event, does not provide information about the cause. A change in ISP complexity should alert the clinician to investigate list of possible causes e.g. increased cord swelling (high ISP), reduced cord perfusion (low SCPP), more deranged autoregulation (high sPRx) and perhaps fever, pneumonia, hypoxia etc. To make our findings more clinically relevant, future studies ought to determine if α, MSE, and λ_max_ are affected by confounding factors unrelated to the injury and ought to validate these metrics in a larger group of TSCI patients and against other outcome measures, not only AIS conversion. Provided these limitations are addressed, α, MSE and λ_max_ may be used clinically. The first step is to incorporate them into a multi-modality display in ICU by computing them in real time perhaps using a sliding 4-h window updated each minute (15, 31, 33). This will allow use of α, MSE, and λ_max_ to evaluate the state of the injury site and the effectiveness of therapies. An alternative to multi-modality monitoring is to combine several ISP-related parameters (e.g., ISP amplitude, SCPP, sPRx, α, MSE, λ_max_) after appropriate weighting into a single, composite metric (cord complexity index) that reflects the state of the injury site. The cord complexity index could also be used to predict outcome independent of the AIS grade. Our observation that non-linear ISP signal metrics carry clinically useful information raises the intriguing possibility of novel therapies for acute TSCI aiming to enhance α, MSE, and λ_max_ without necessarily reducing ISP or increasing SCPP.

Further research is required to fully define the clinical value of non-linear analysis of physiological signals, such as intracranial or intraspinal pressure monitored from injured brain or spinal cord in ICU. To facilitate this, we recommend that non-linear metrics be incorporated into standard software packages that are widely used by neuro-ICUs, such as ICM+.

## Author contributions

SC wrote software and analyzed the data to produce alpha, multiscale entropy, and lambda values. MG collected and cleaned the raw data and patient demographics. MP did the surgical procedures, looked after the patients, and inserted the probes. MP and SS analyzed data, produced the figures and wrote the manuscript. SS supervised the project.

### Conflict of interest statement

The authors declare that the research was conducted in the absence of any commercial or financial relationships that could be construed as a potential conflict of interest.

## Abbreviations

AIS: American spinal injuries association Impairment Scale
Alpha (α): exponent from detrended fluctuation analysis
DFA: detrended fluctuation analysis
ICU: intensive care unit
ISCoPE: injured spinal cord pressure evaluation study
ISP: intraspinal pressure
Lambda max (λ_max_): maximal Lyapunov exponent
MAP: mean arterial pressure
MSE: multiscale entropy
SCPP: spinal cord perfusion pressure
sPRx: spinal pressure reactivity index
TSCI: traumatic spinal cord injury.
