# Supplementary material for: Non-linear Dynamical Analysis of Intraspinal Pressure Signal Predicts Outcome After Spinal Cord Injury
Source: Front Neurol. 2018 Jun 26;9:493. doi: 10.3389/fneur.2018.00493 (PMC6028604; doi:10.3389/fneur.2018.00493)

## Slide 1
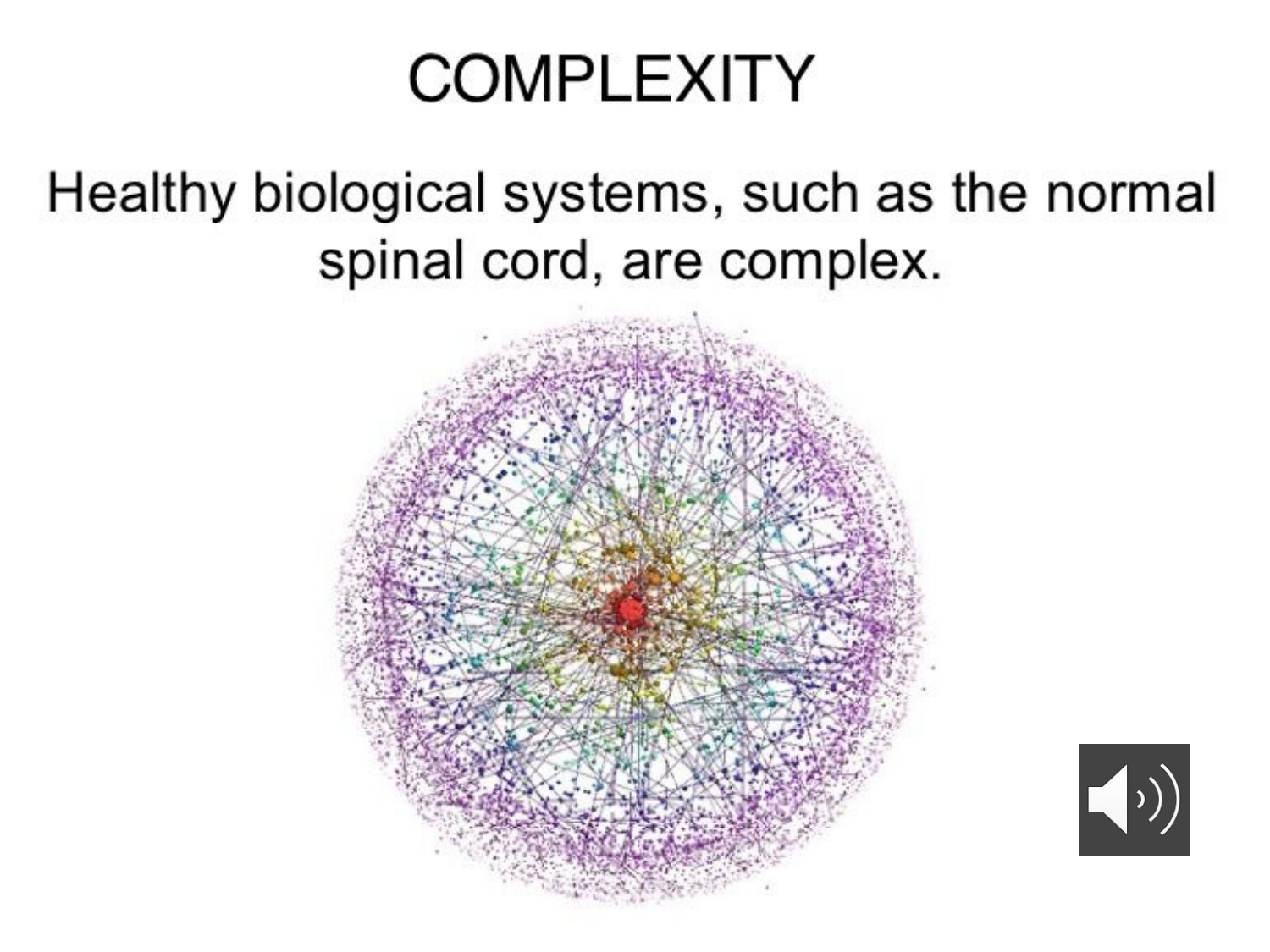

## Slide 2
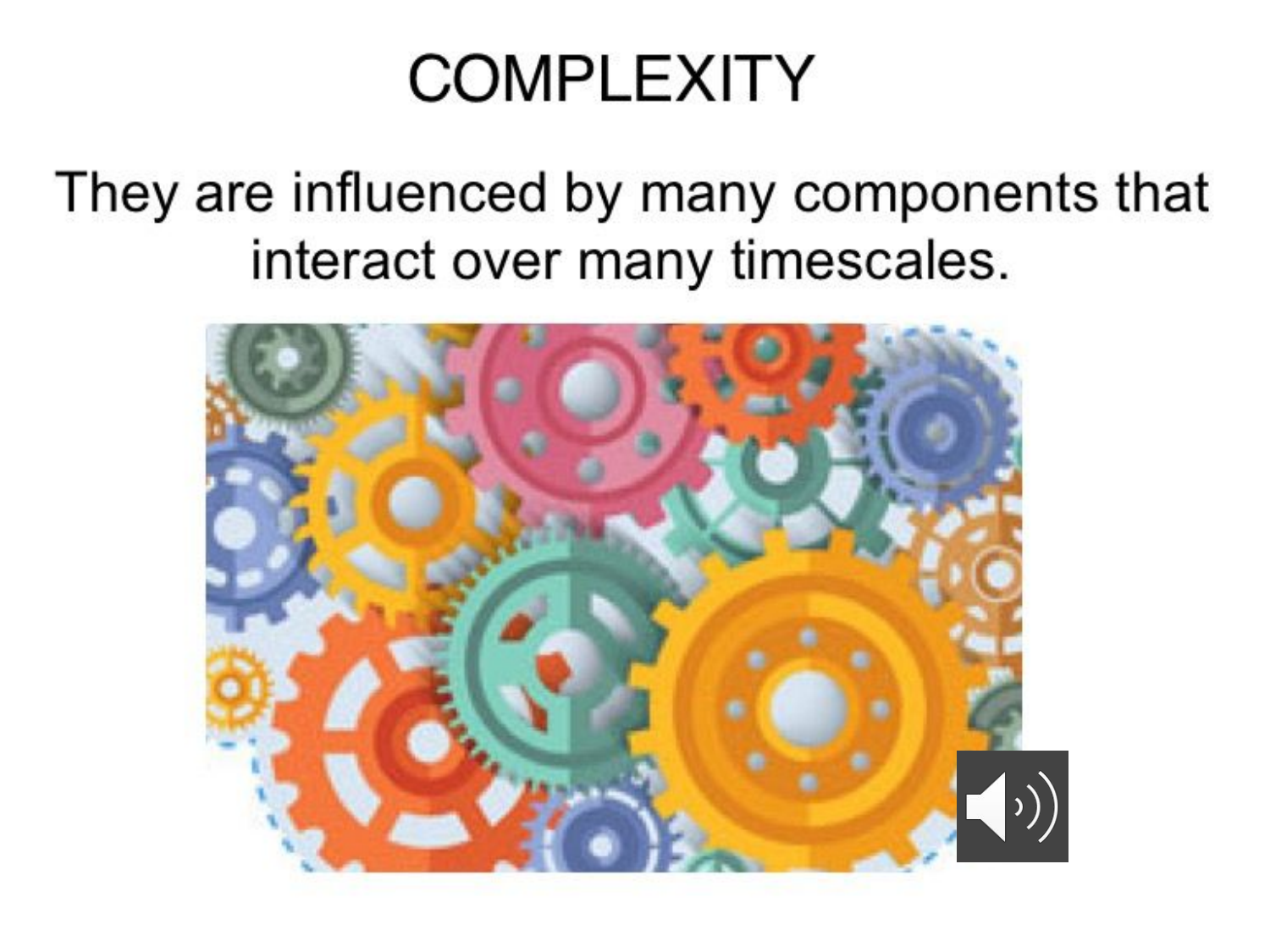

## Slide 3
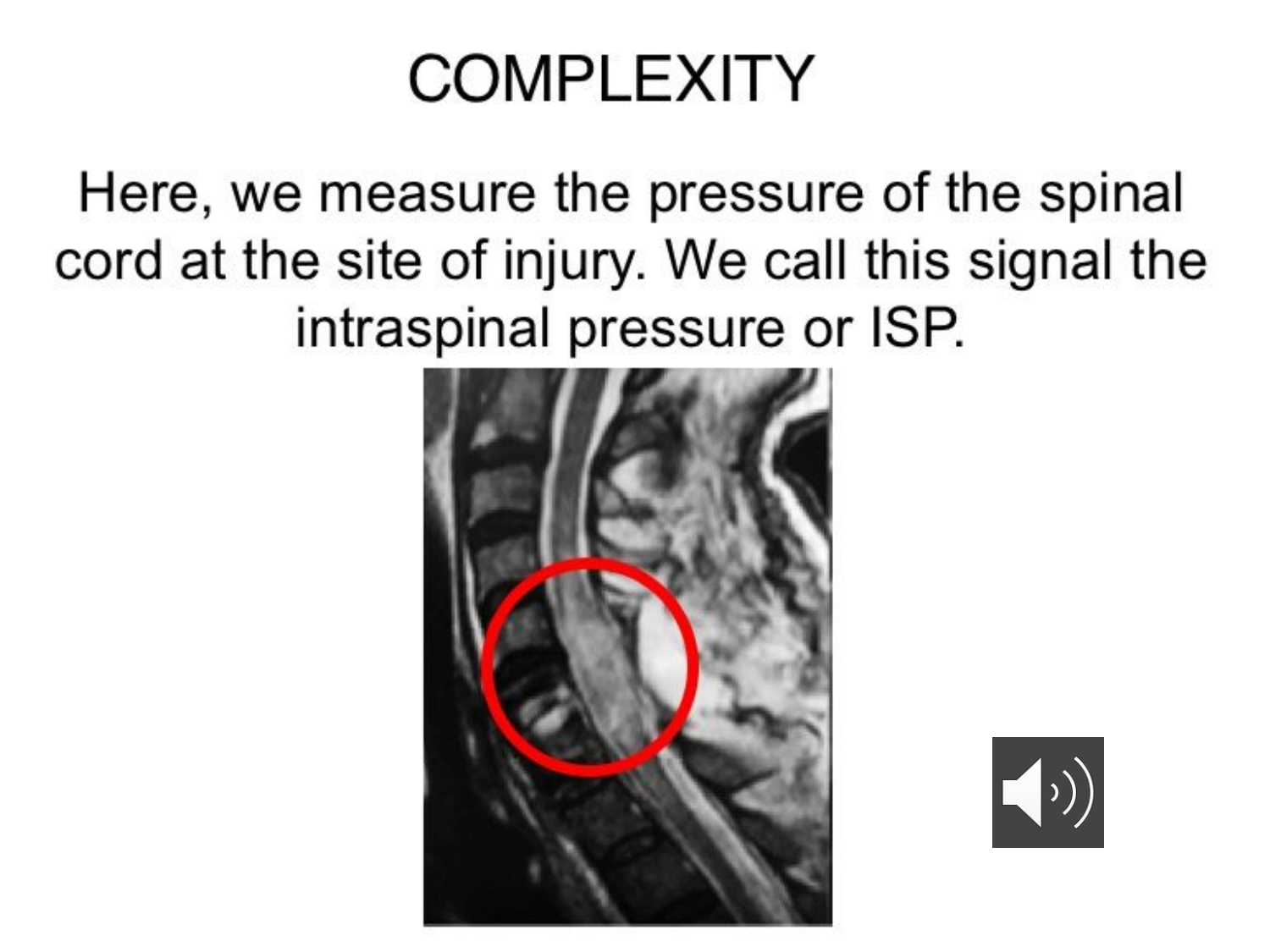

## Slide 4
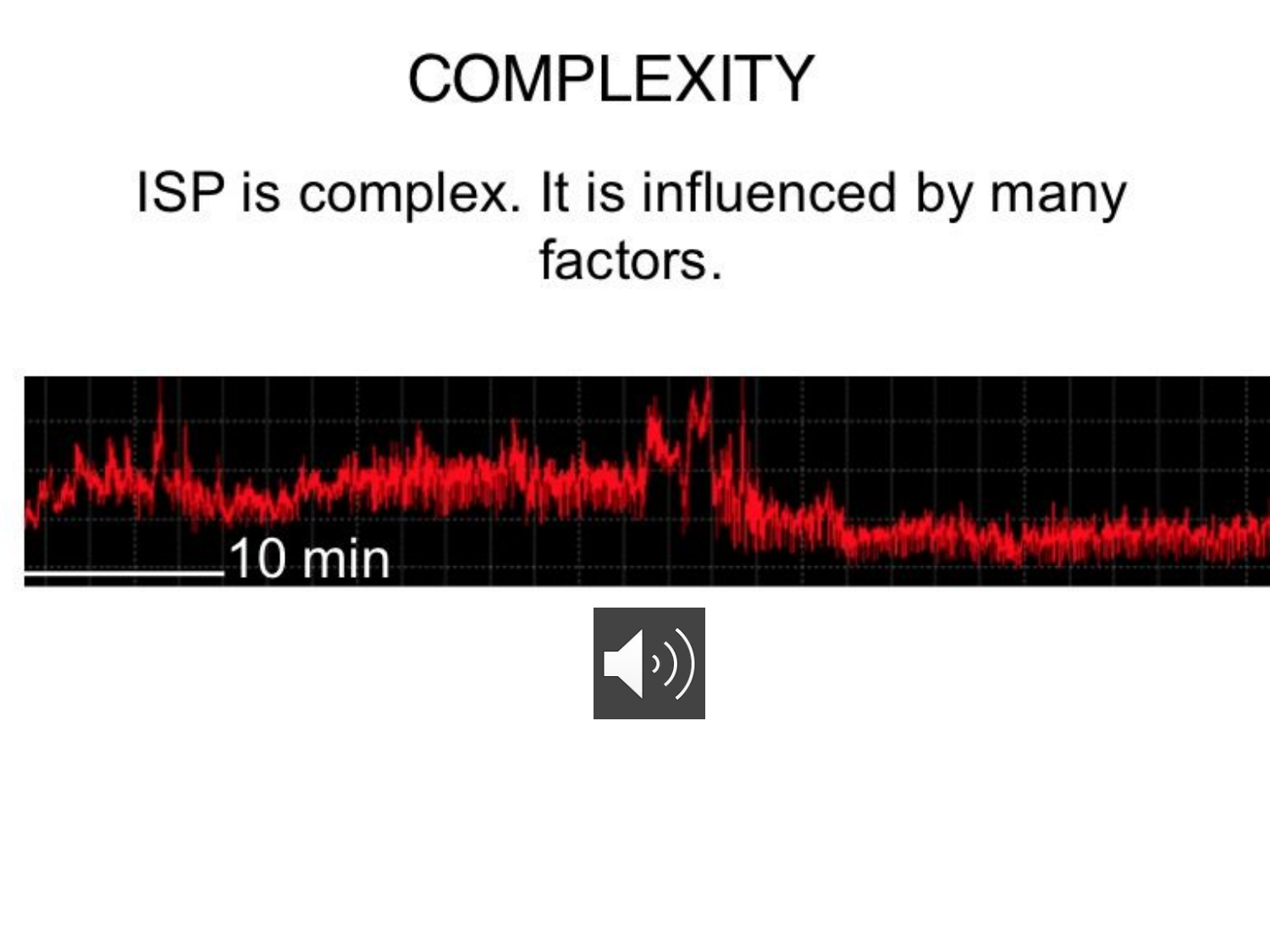

## Slide 5
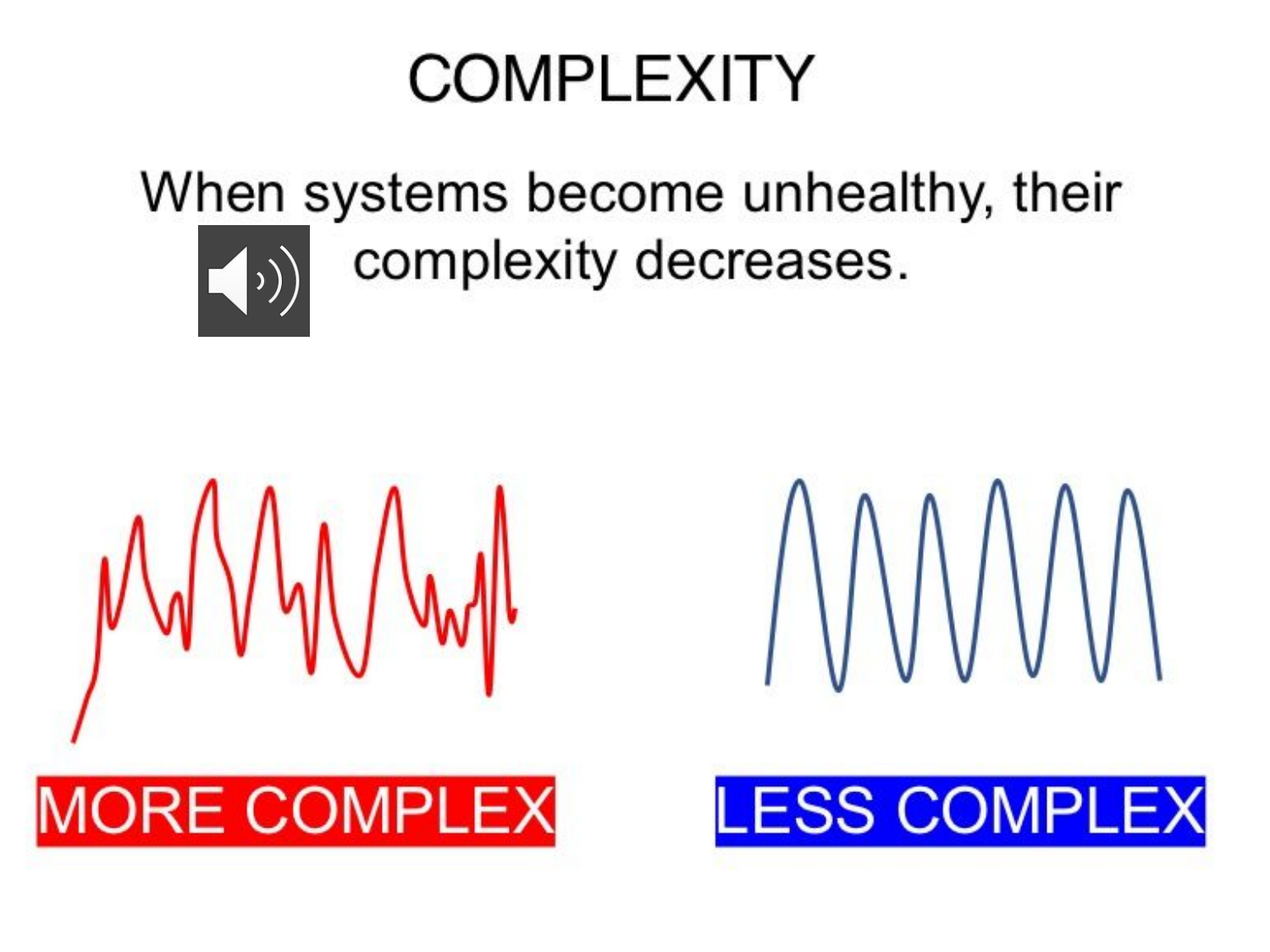

## Slide 6
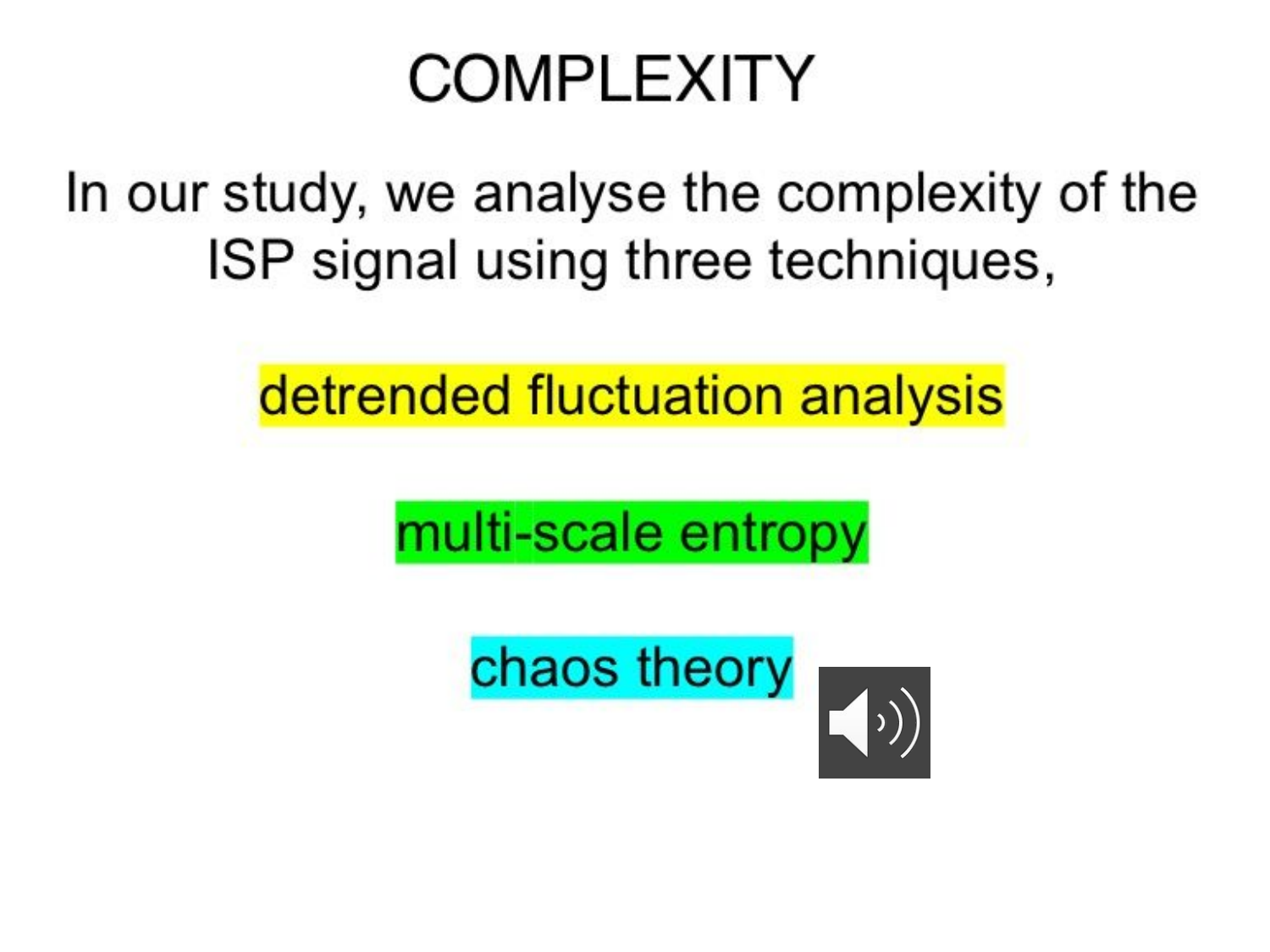

## Slide 7
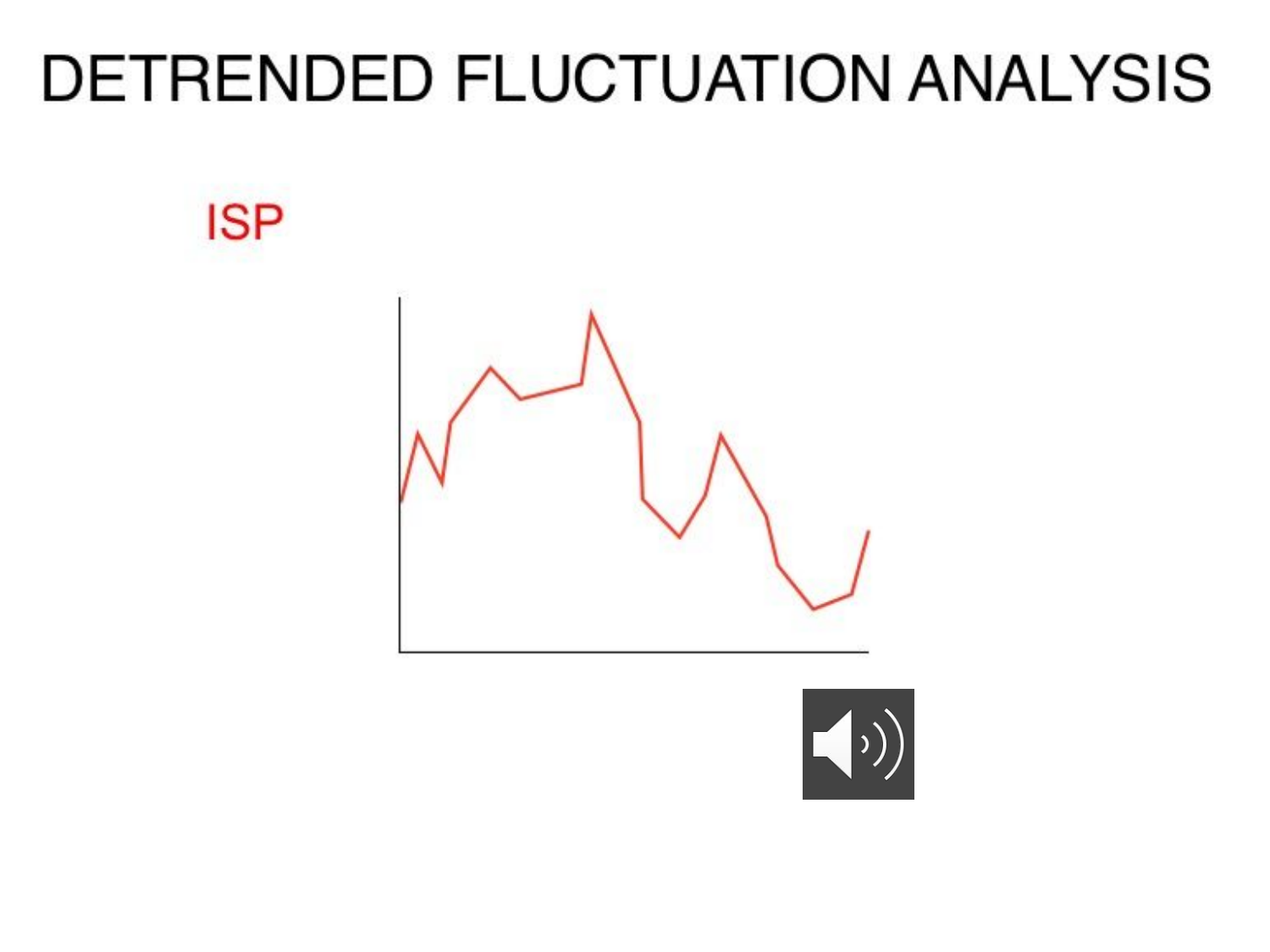

## Slide 8
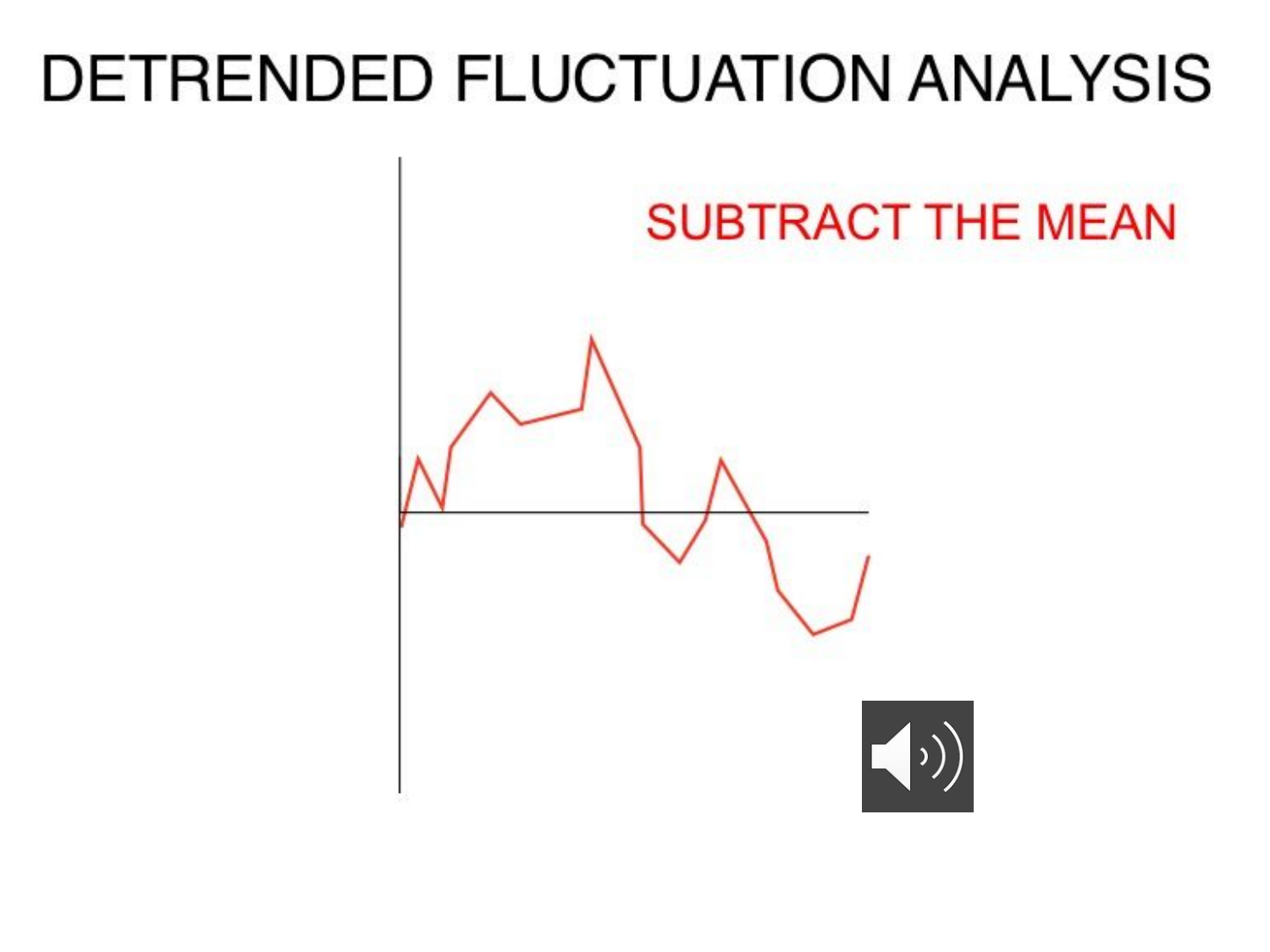

## Slide 9
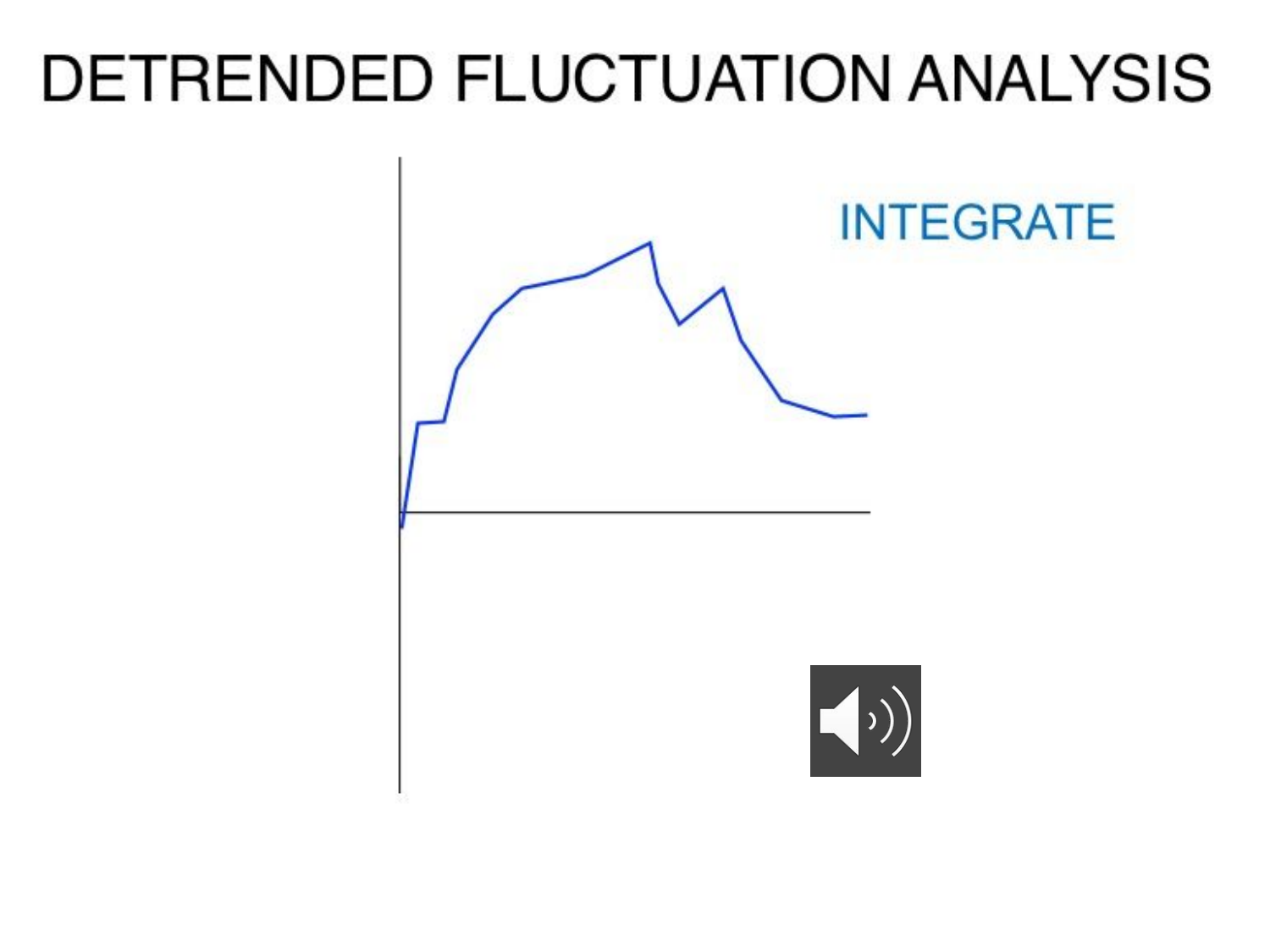

## Slide 10
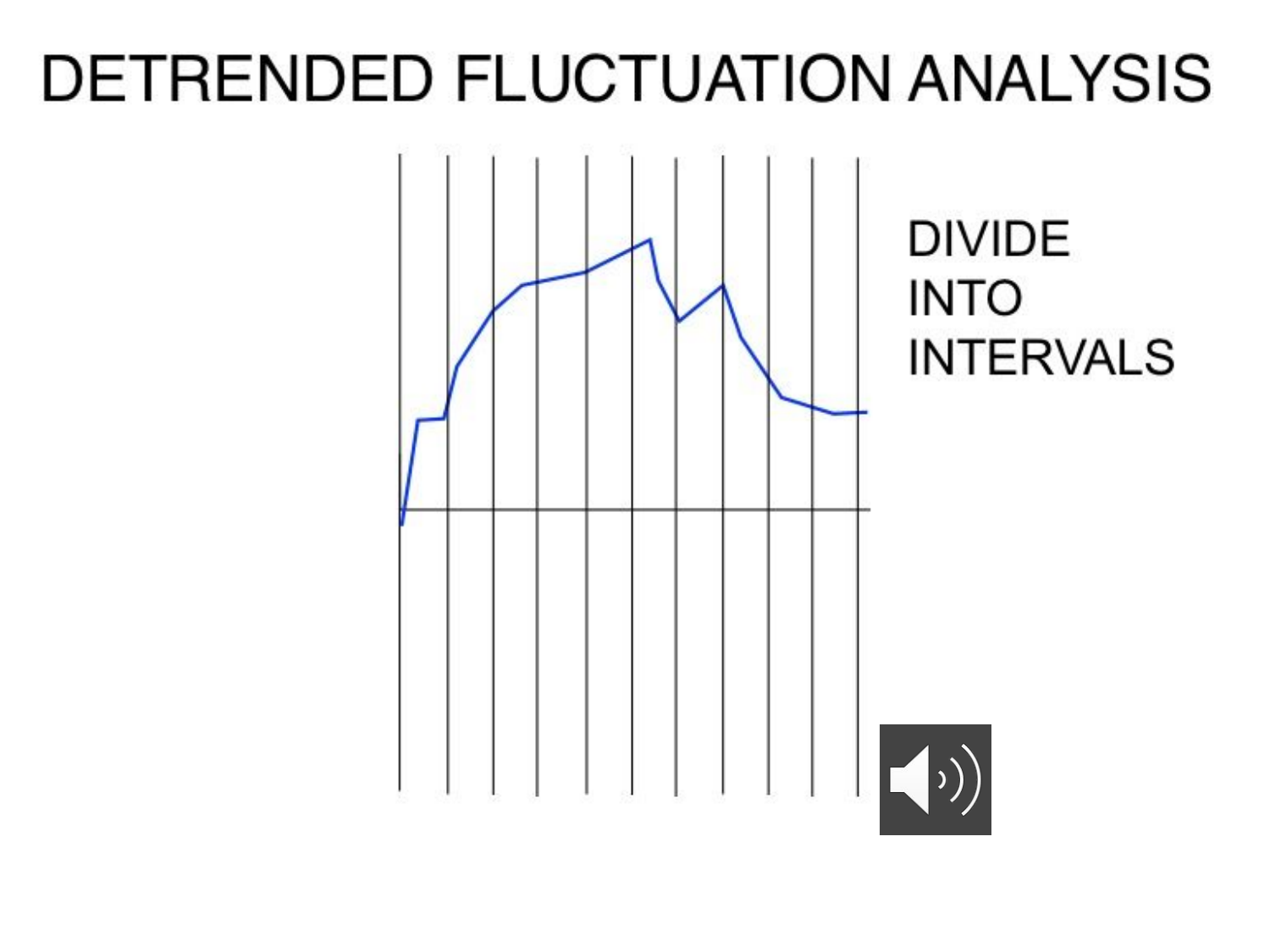

## Slide 11
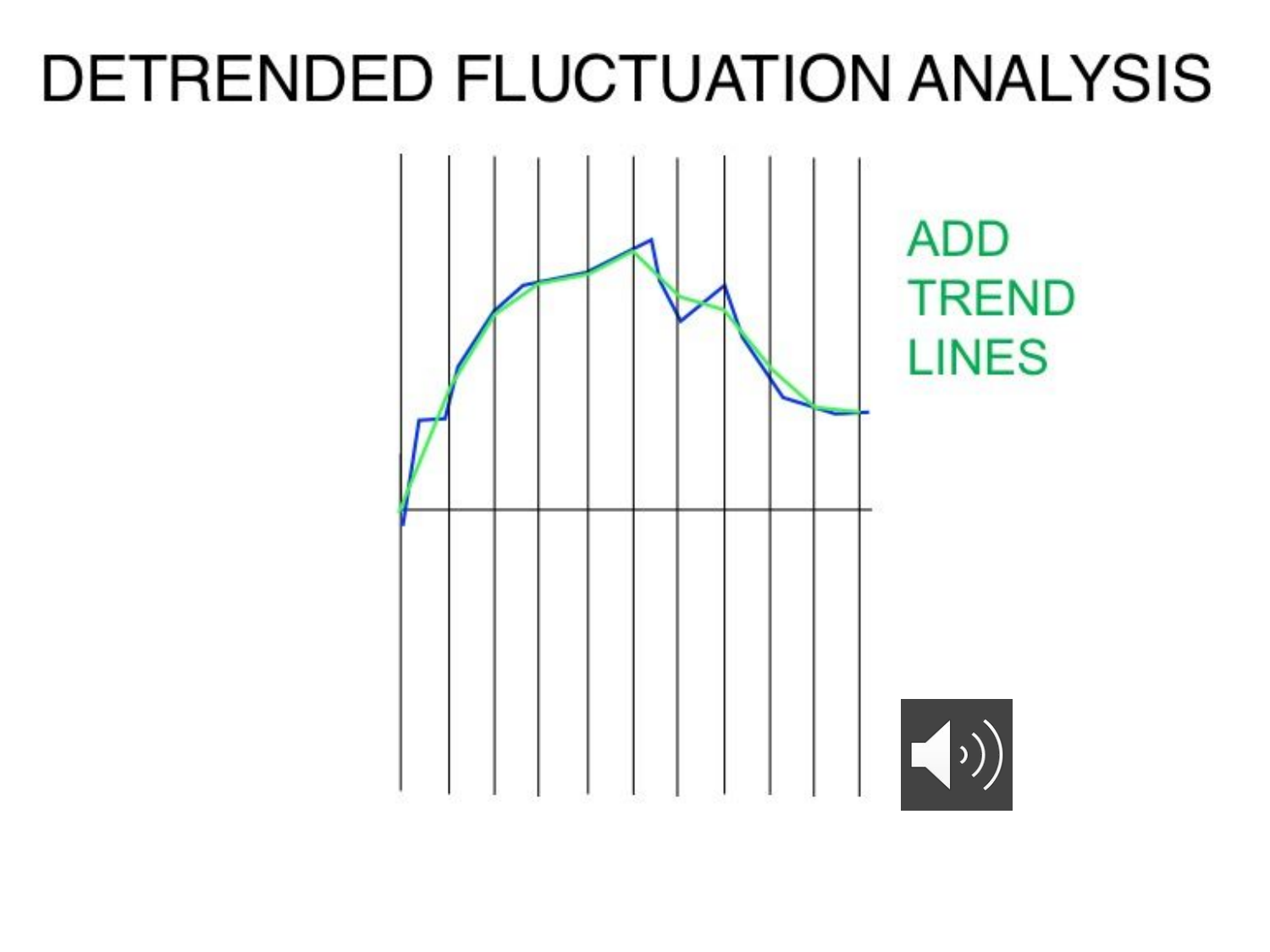

## Slide 12
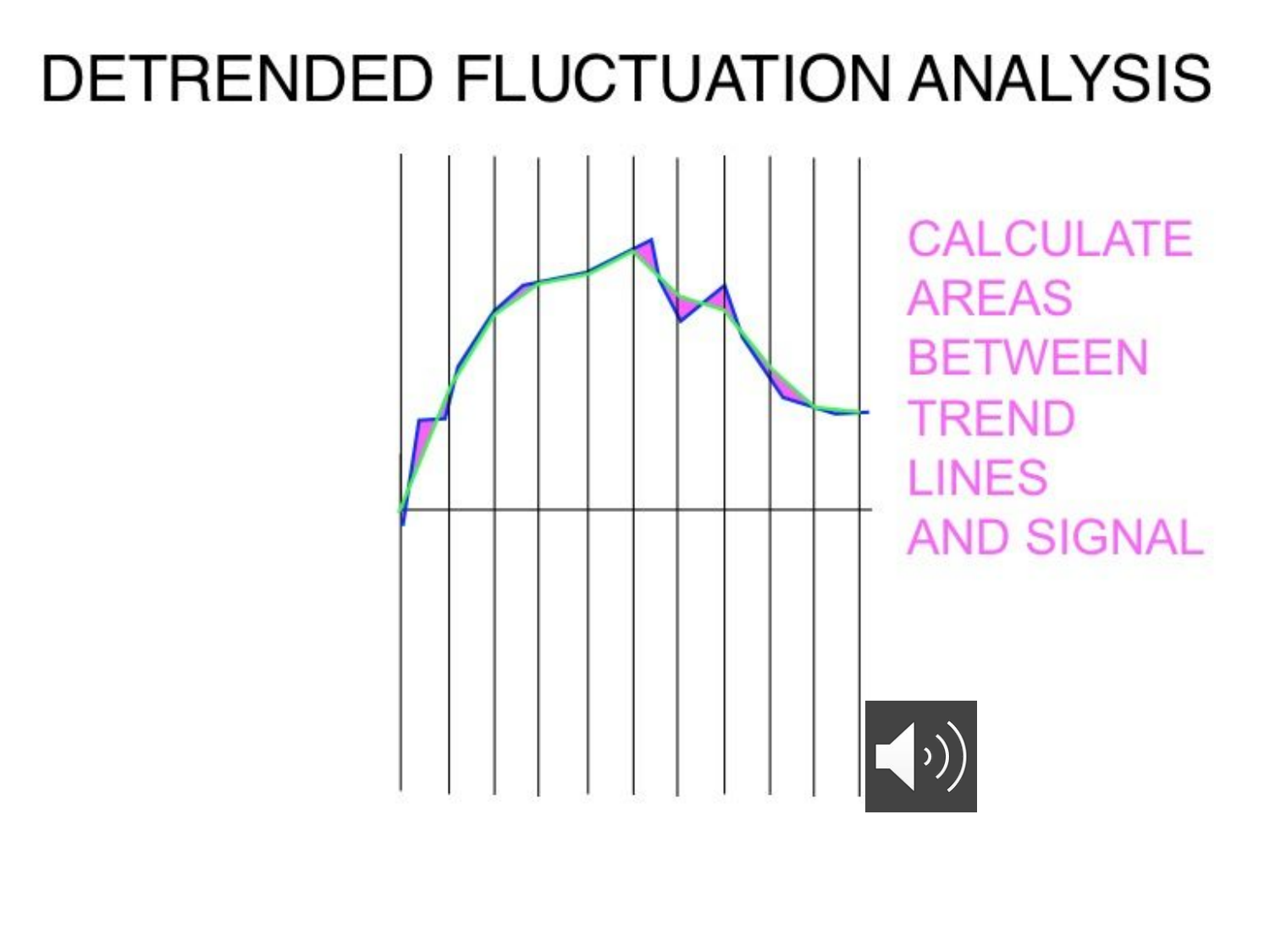

## Slide 13
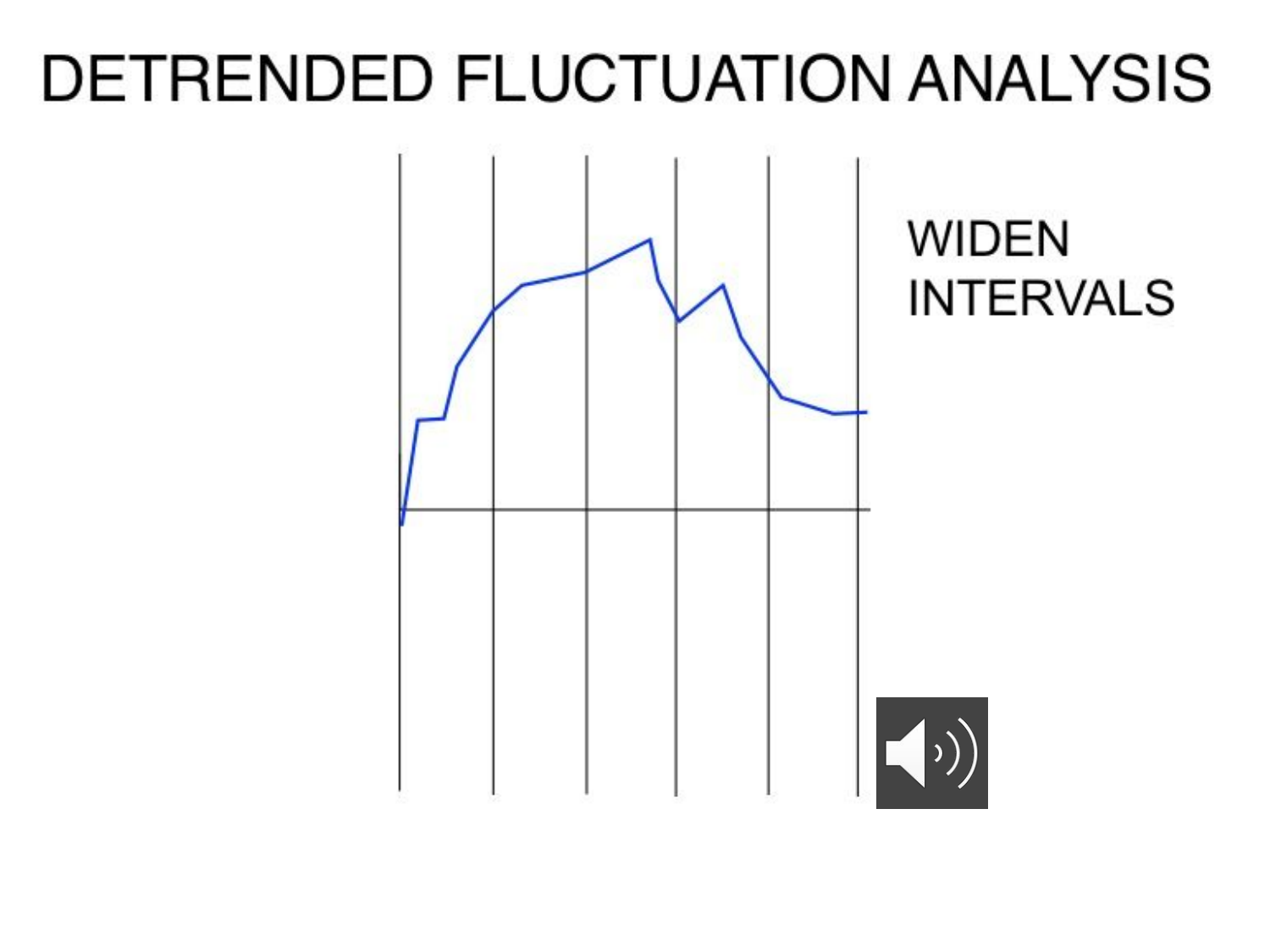

## Slide 14
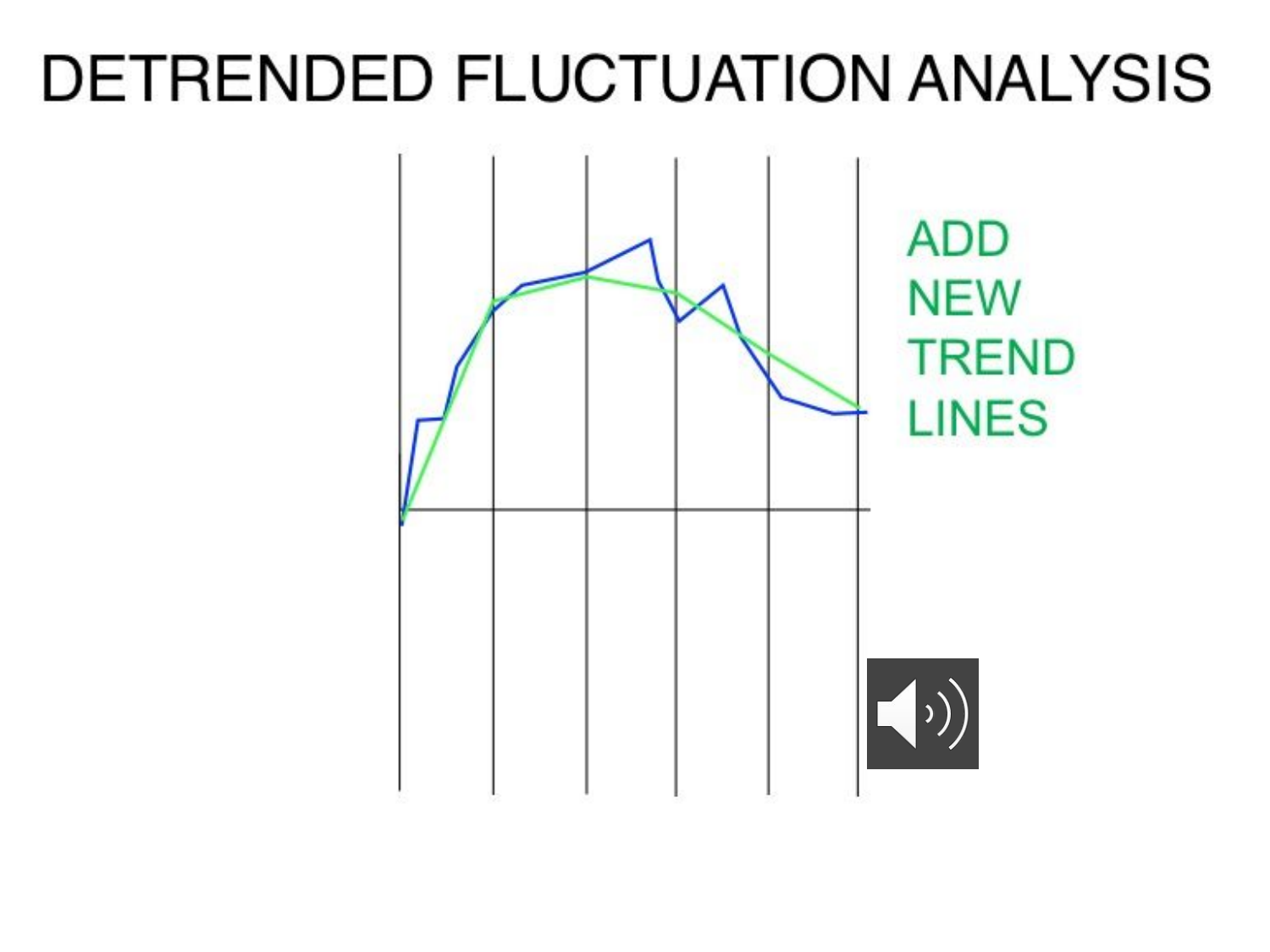

## Slide 15
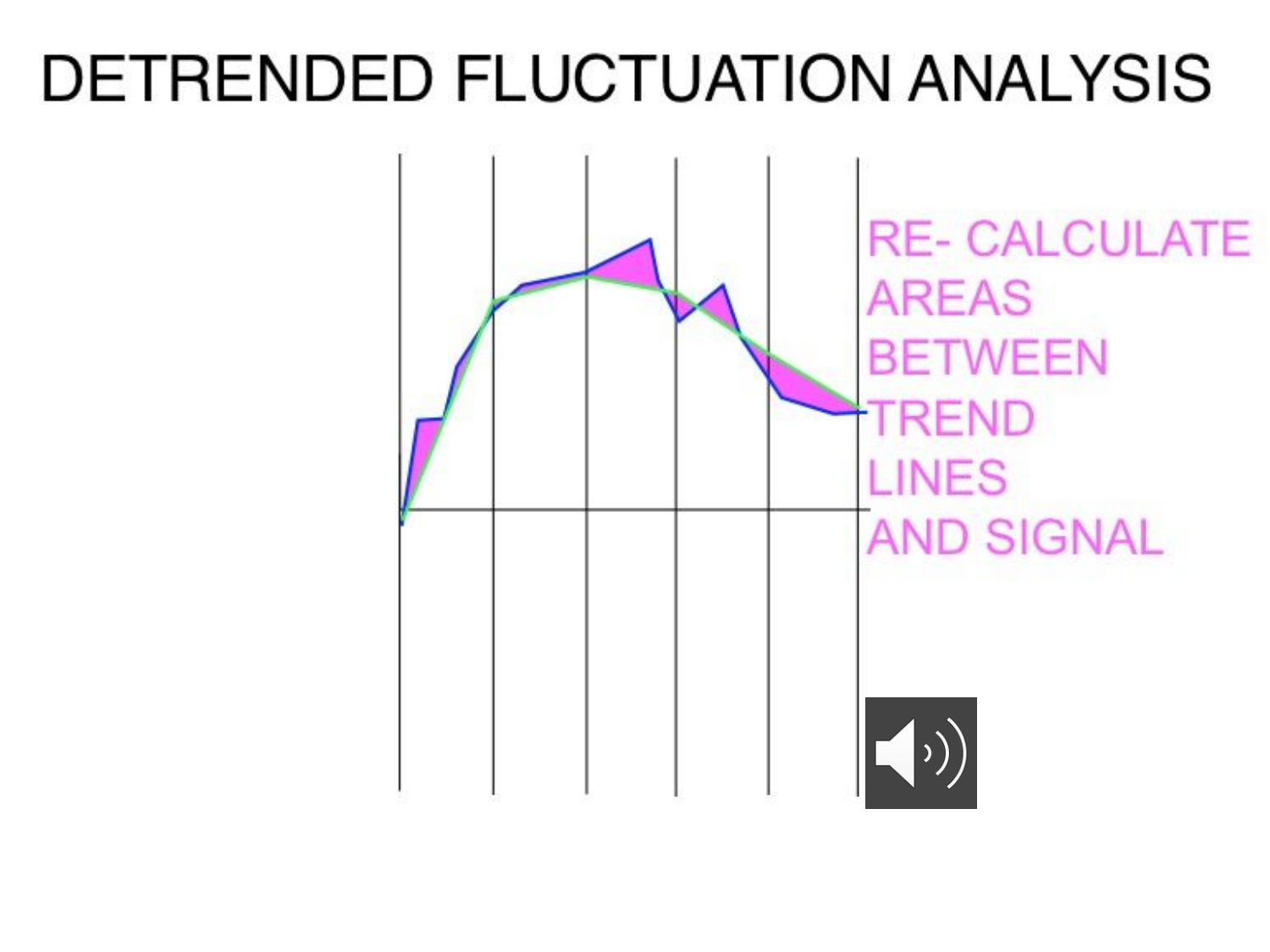

## Slide 16
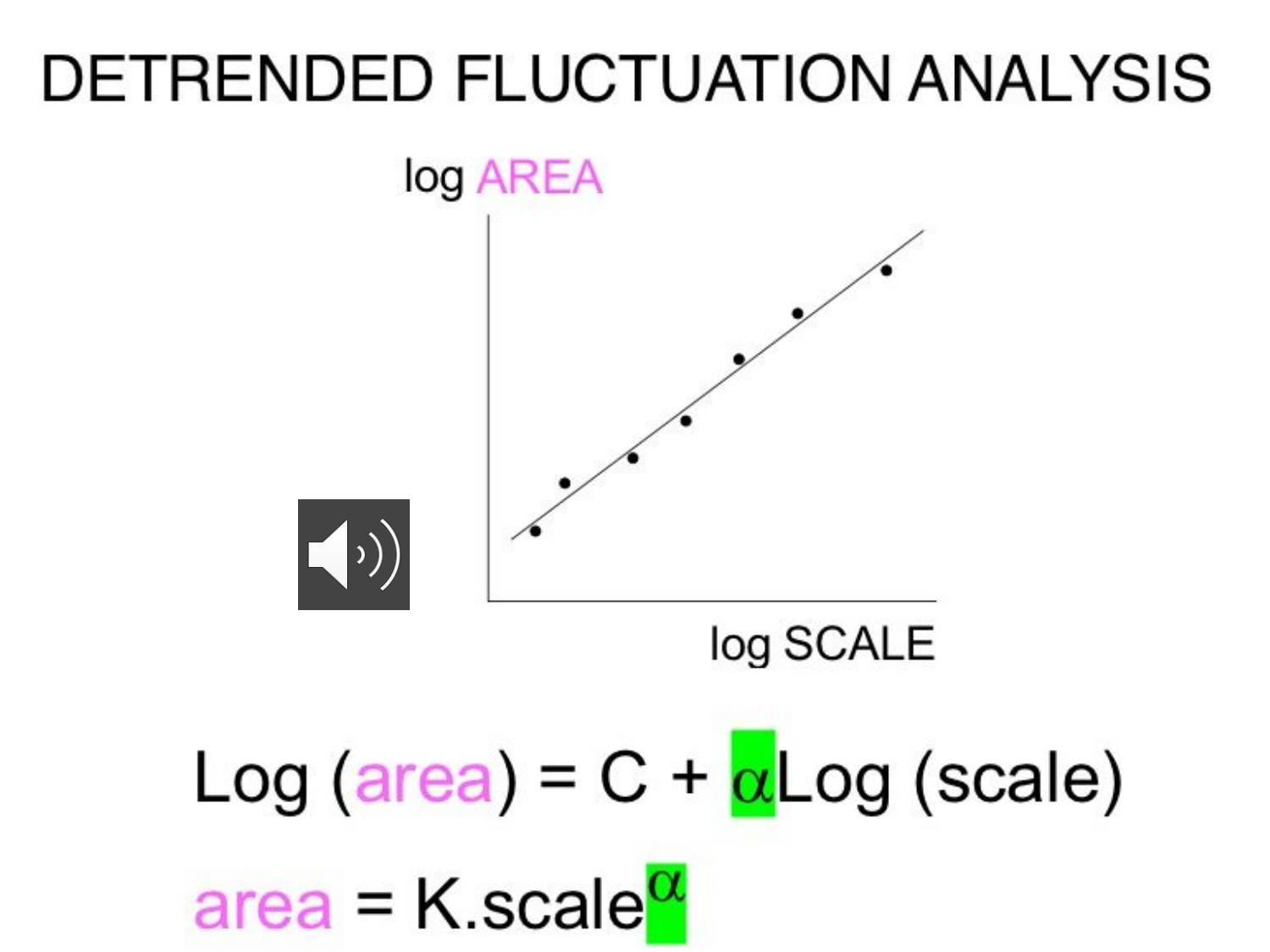

## Slide 17
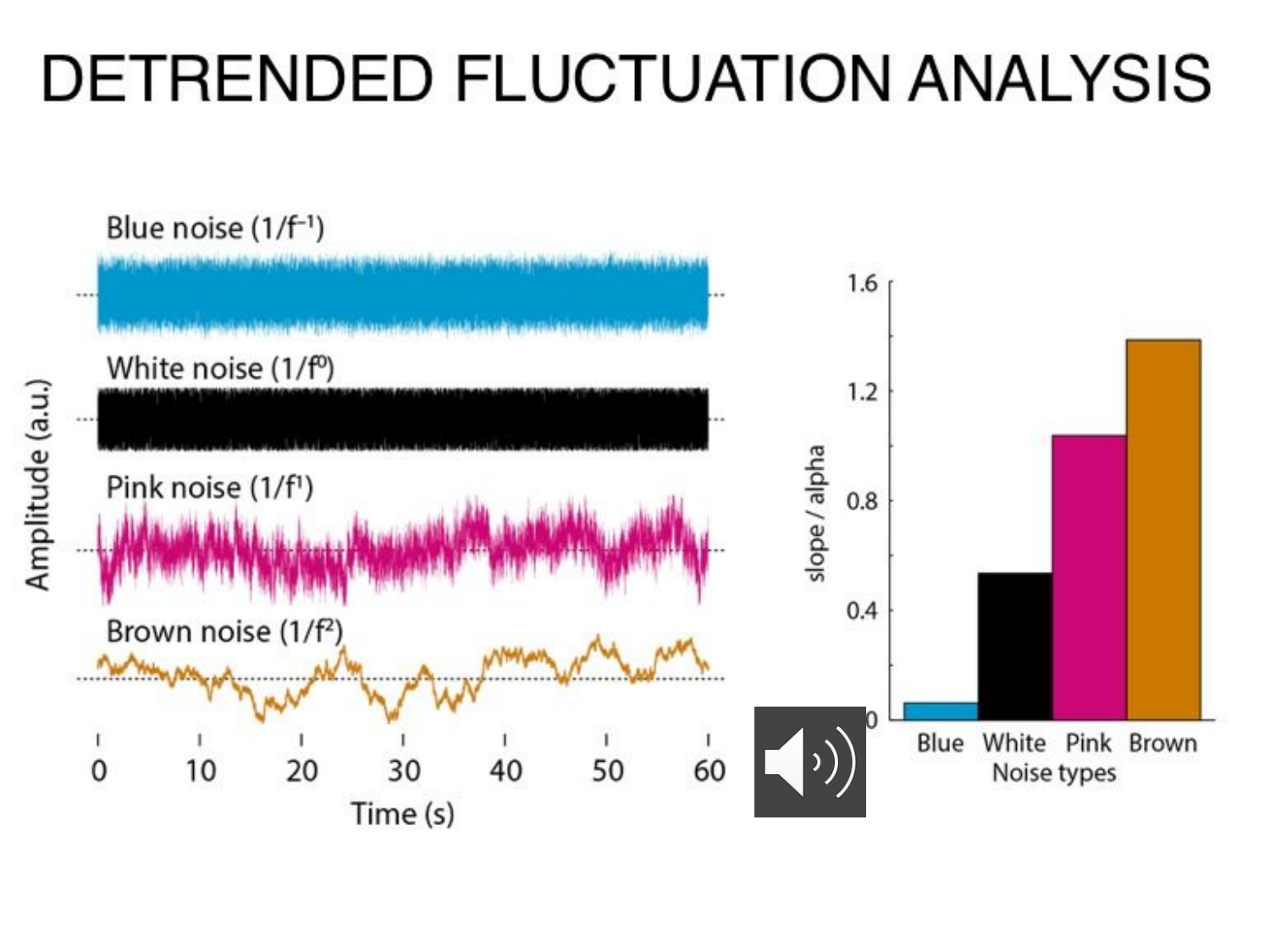

## Slide 18
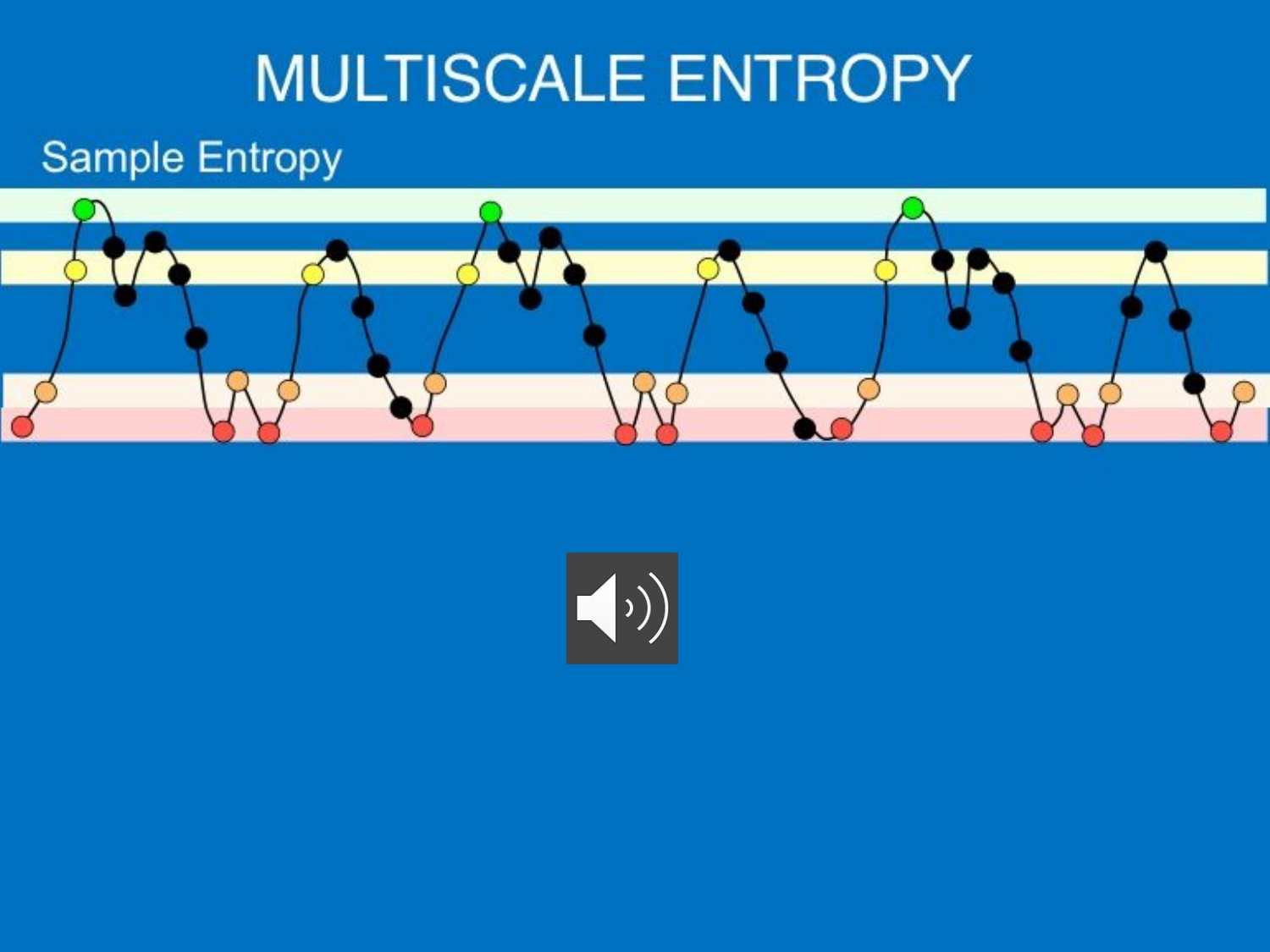

## Slide 19
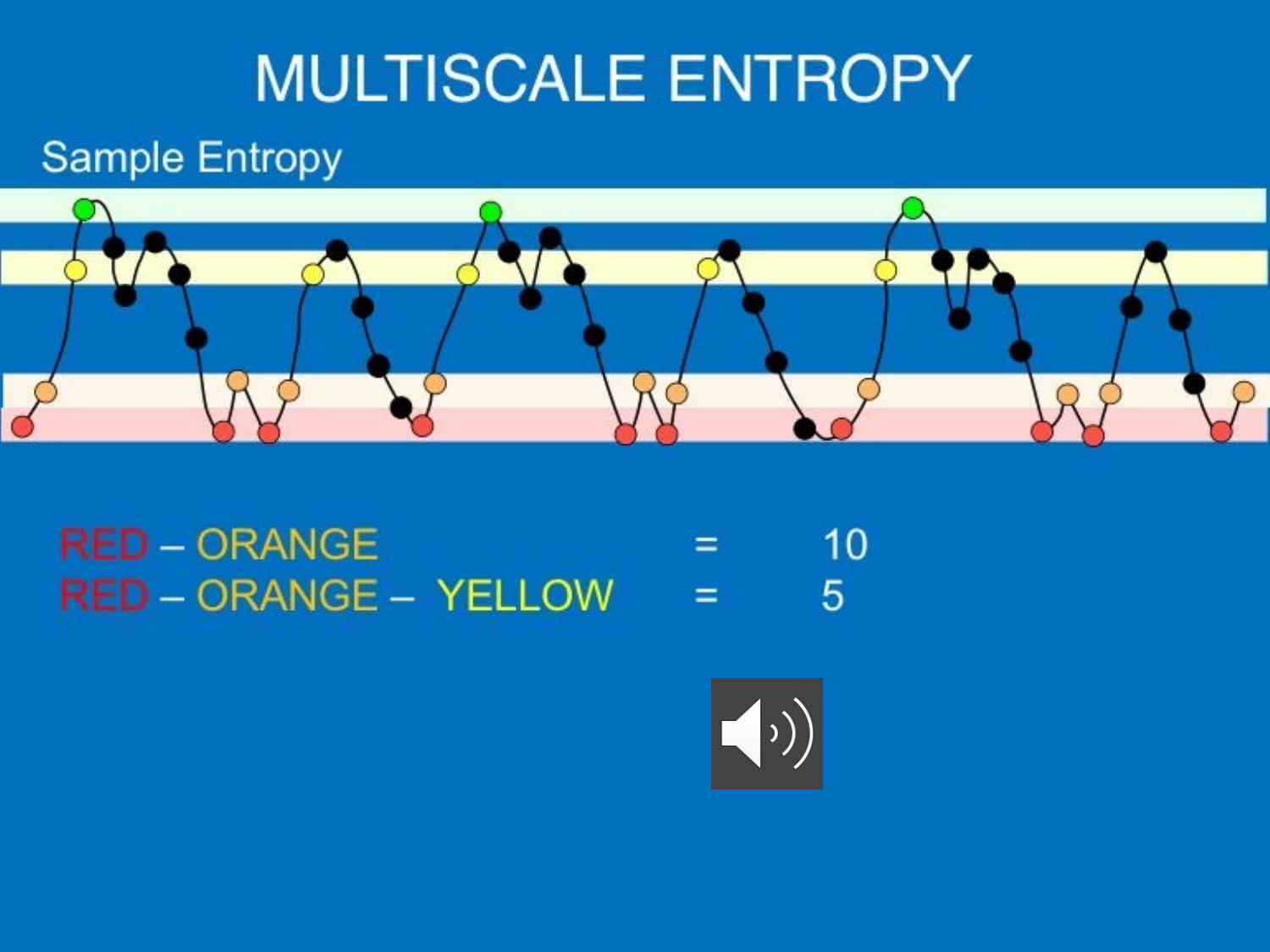

## Slide 20
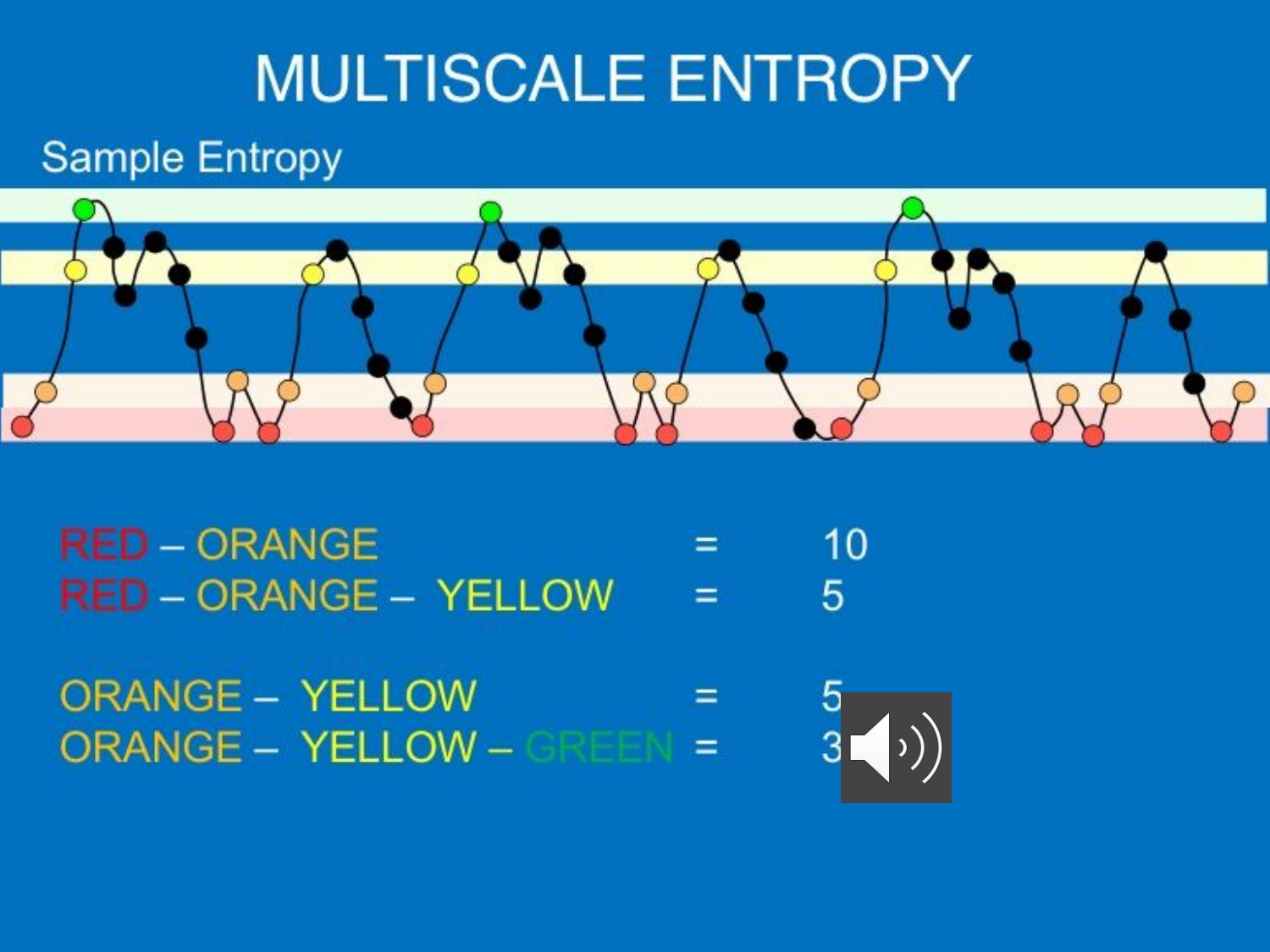

## Slide 21
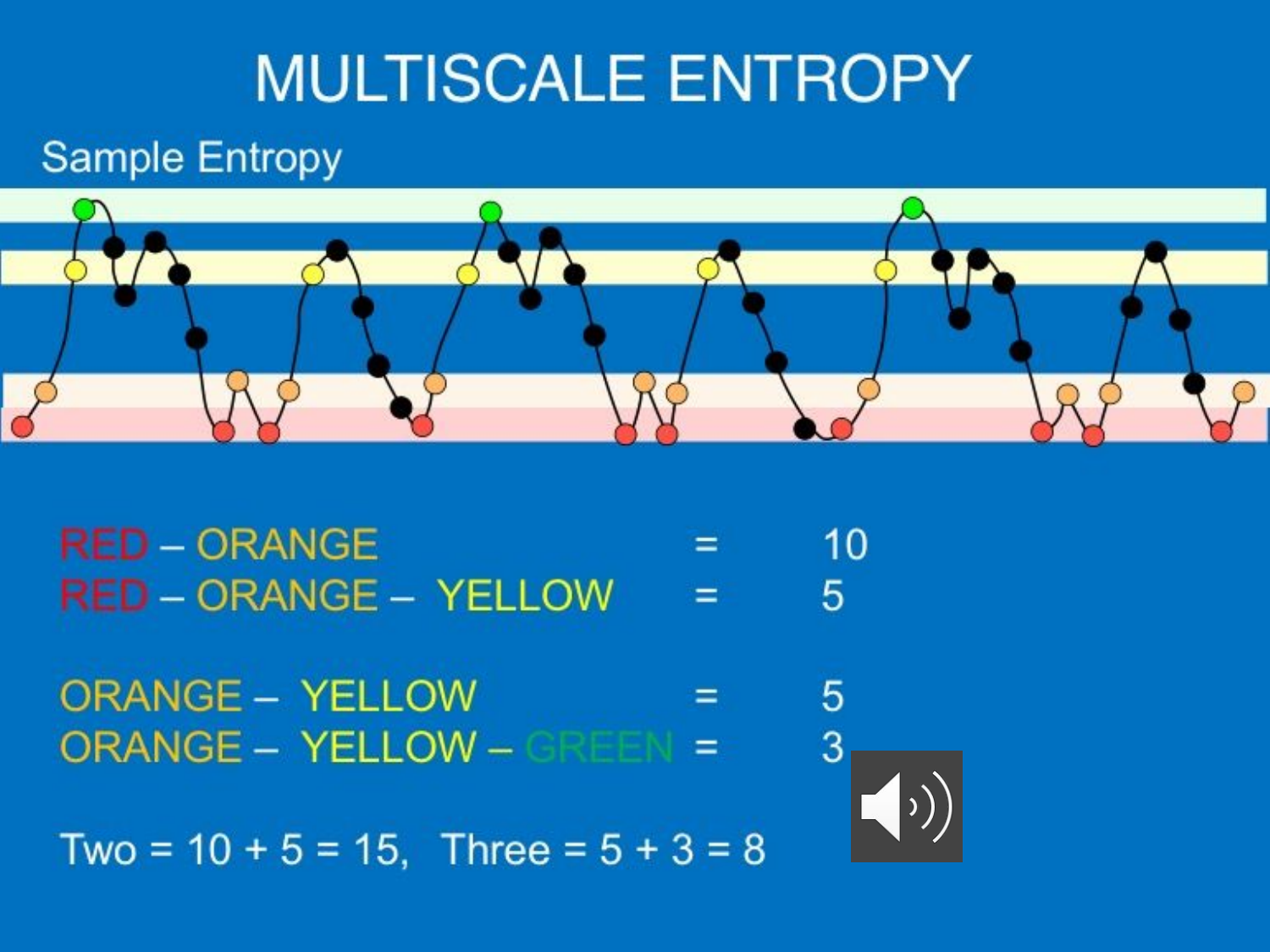

## Slide 22
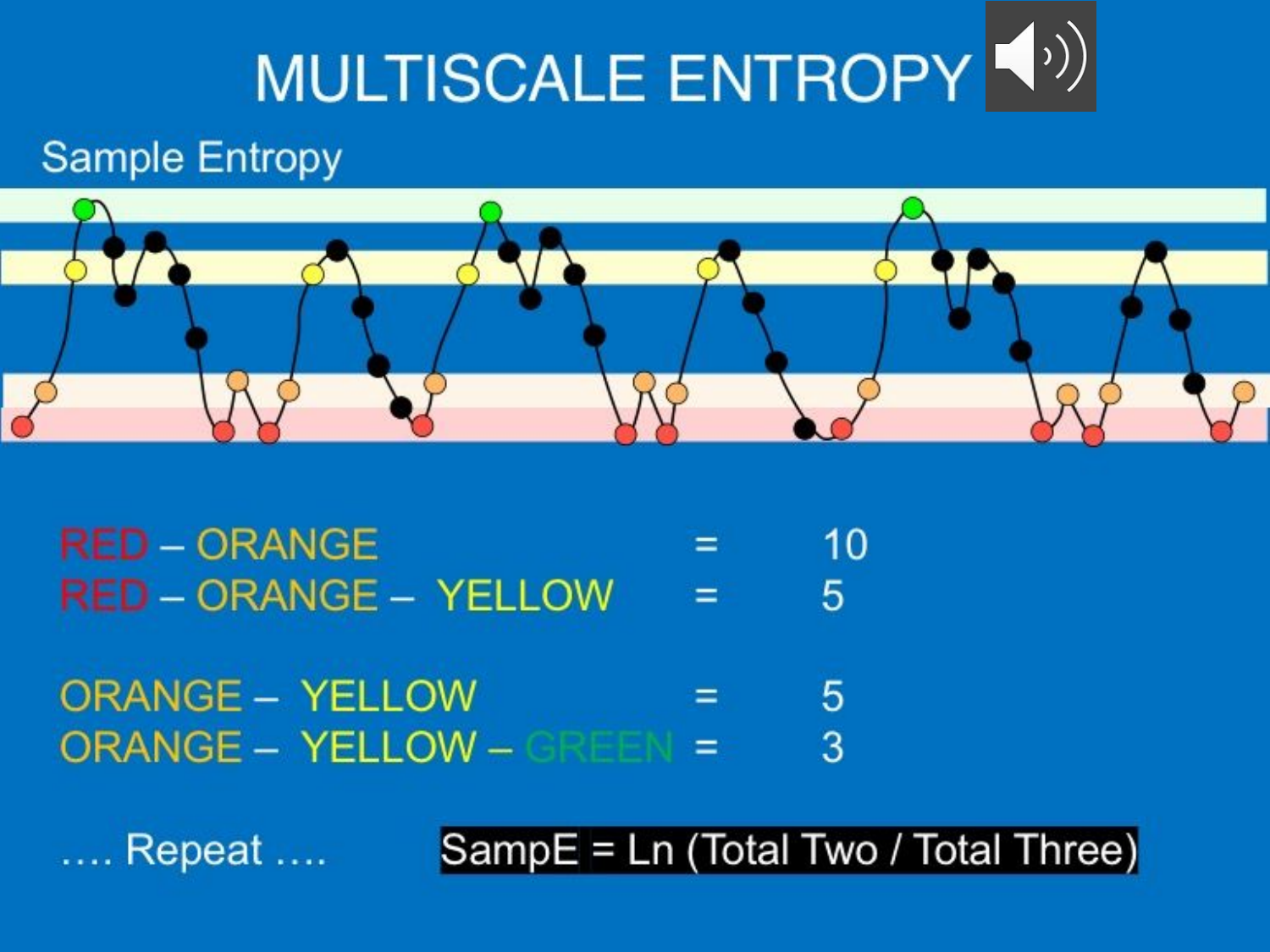

## Slide 23
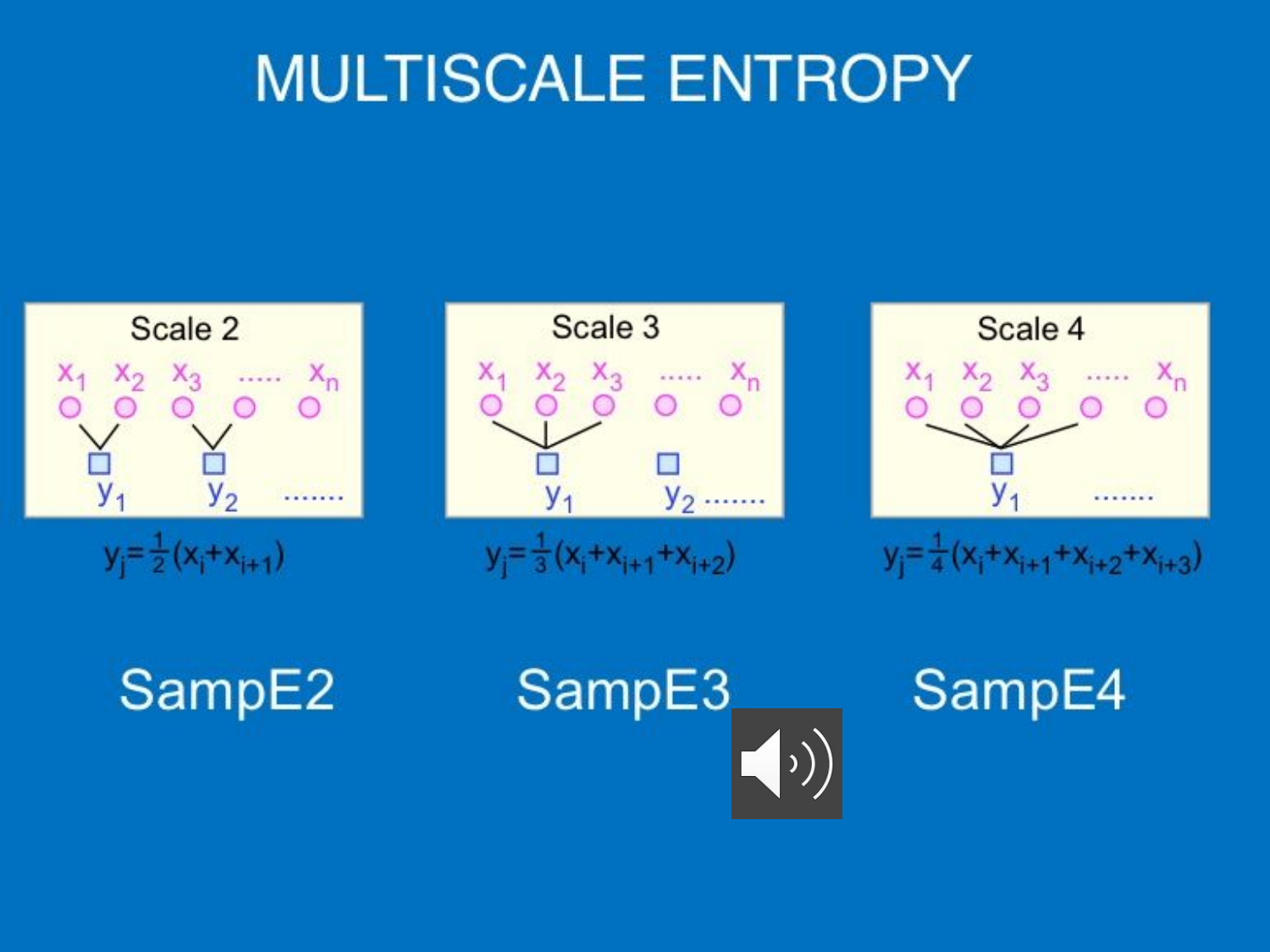

## Slide 24
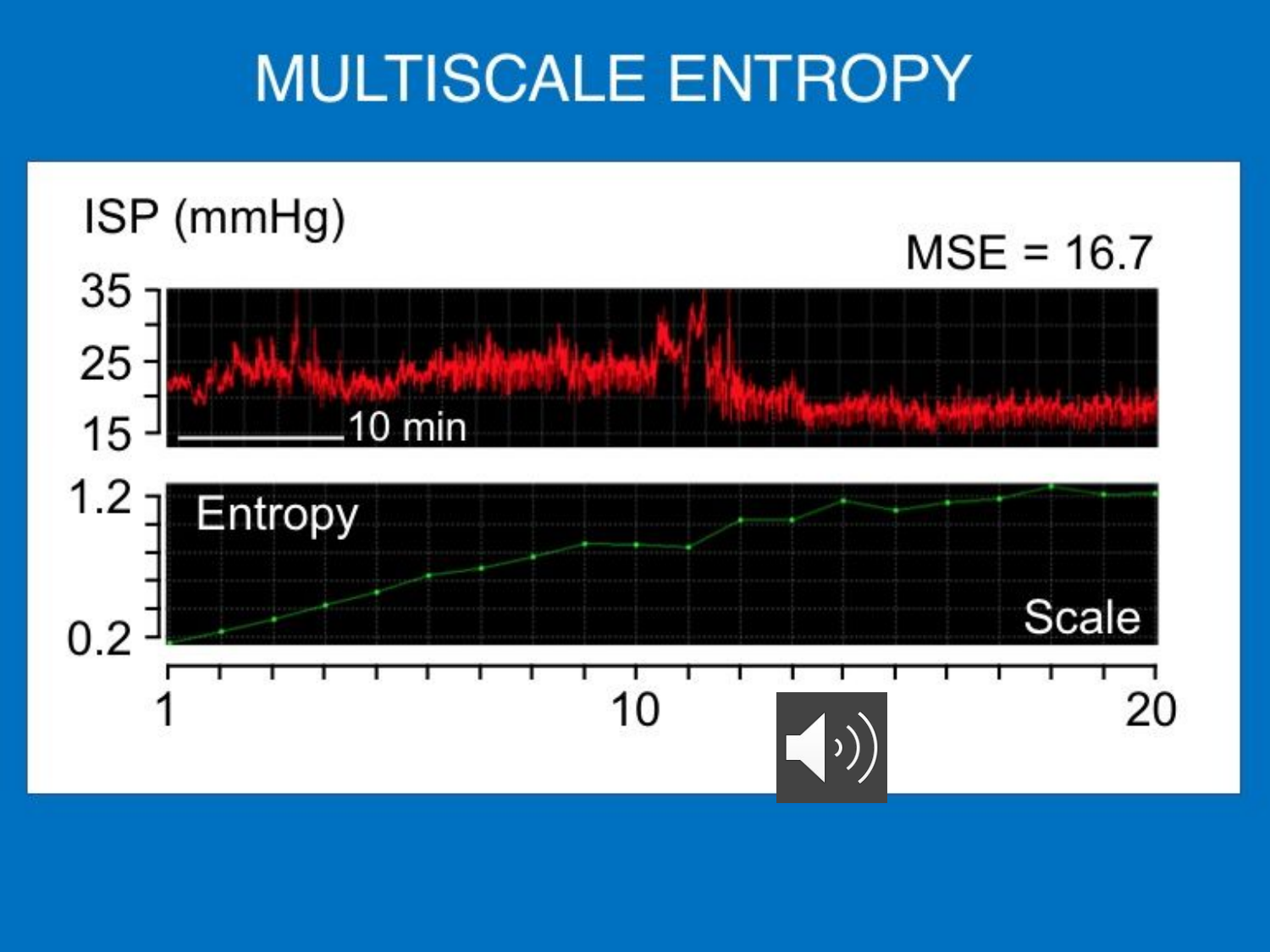

## Slide 25
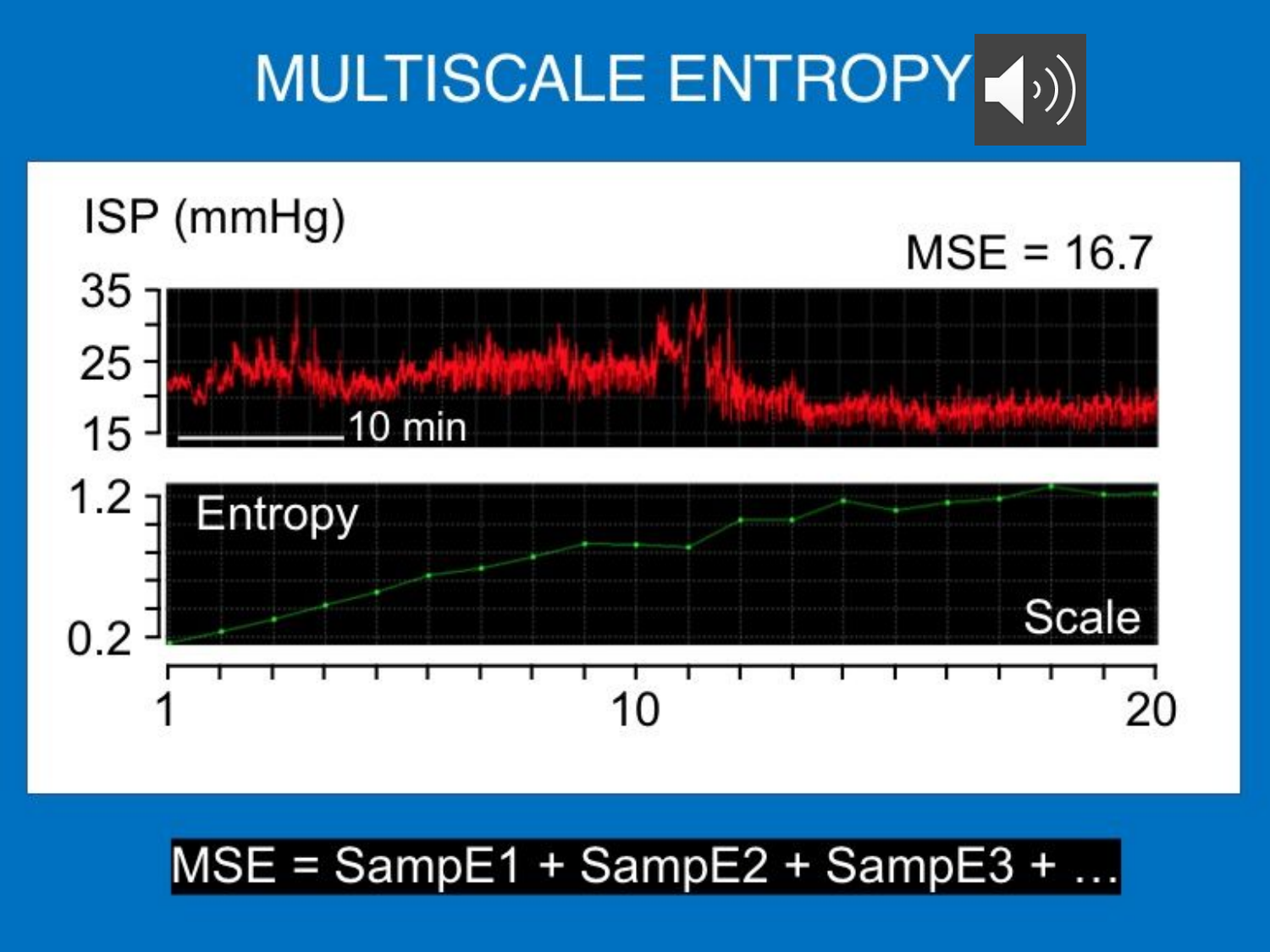

## Slide 26
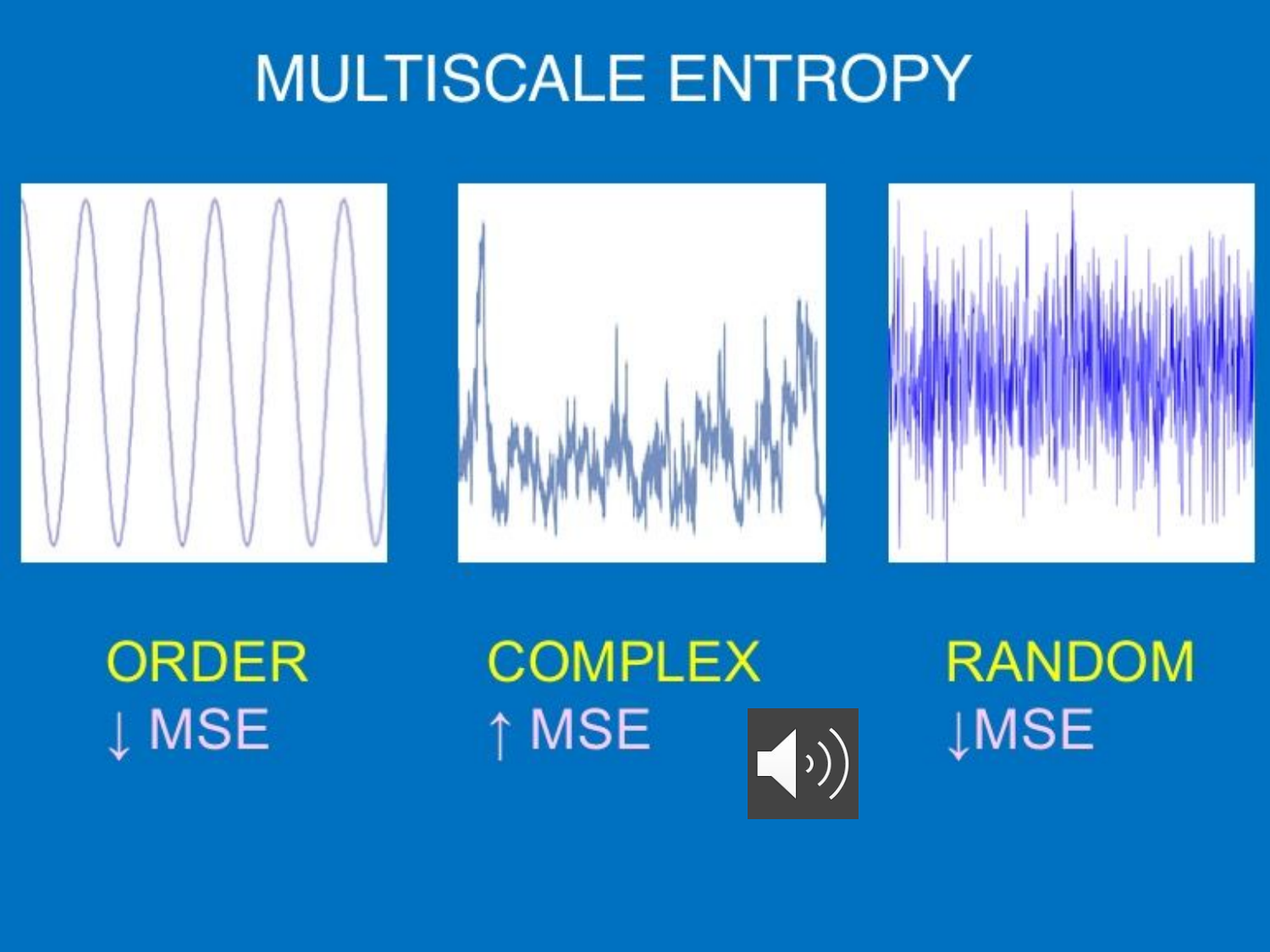

## Slide 27
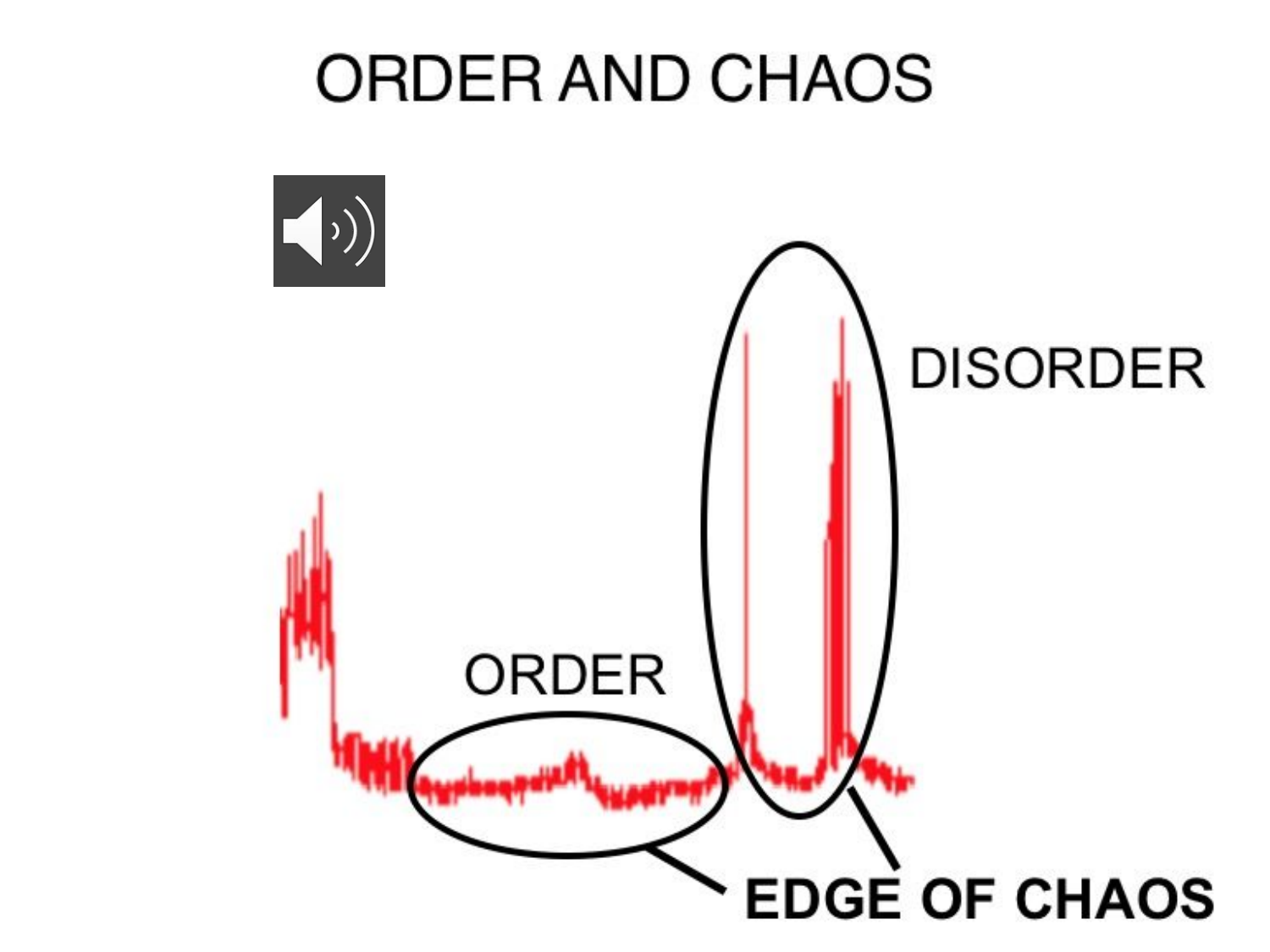

## Slide 28
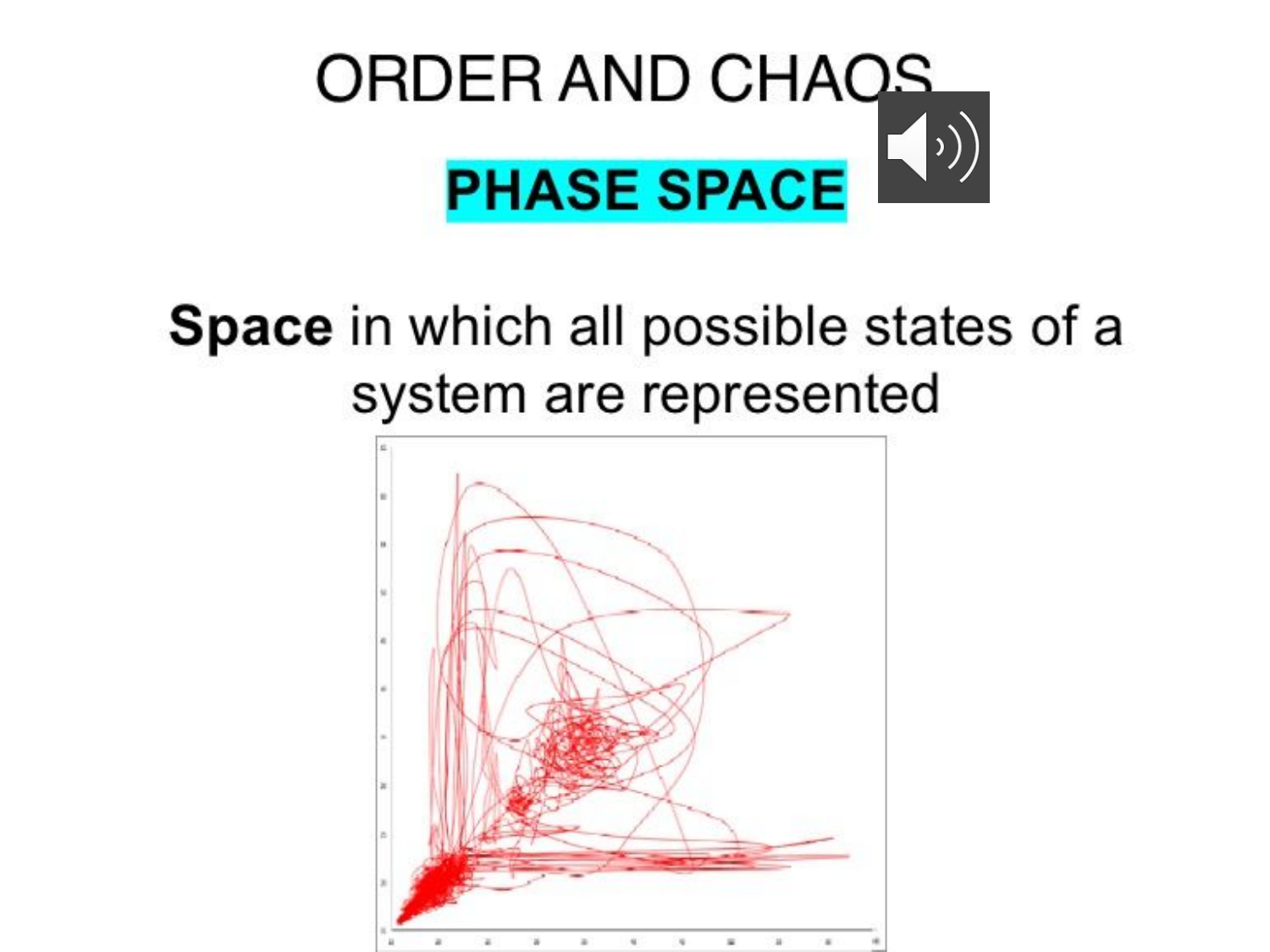

## Slide 29
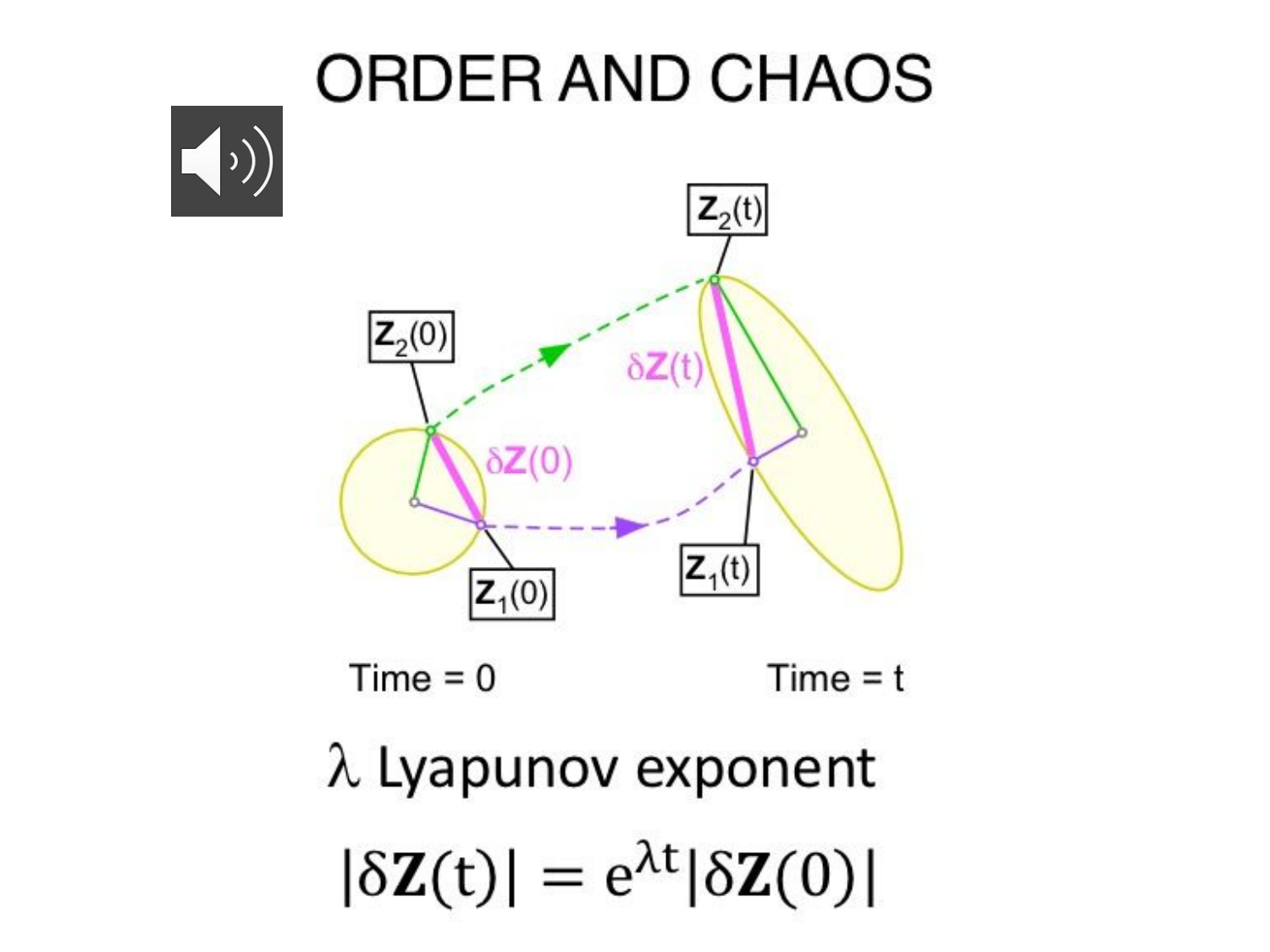

## Slide 30
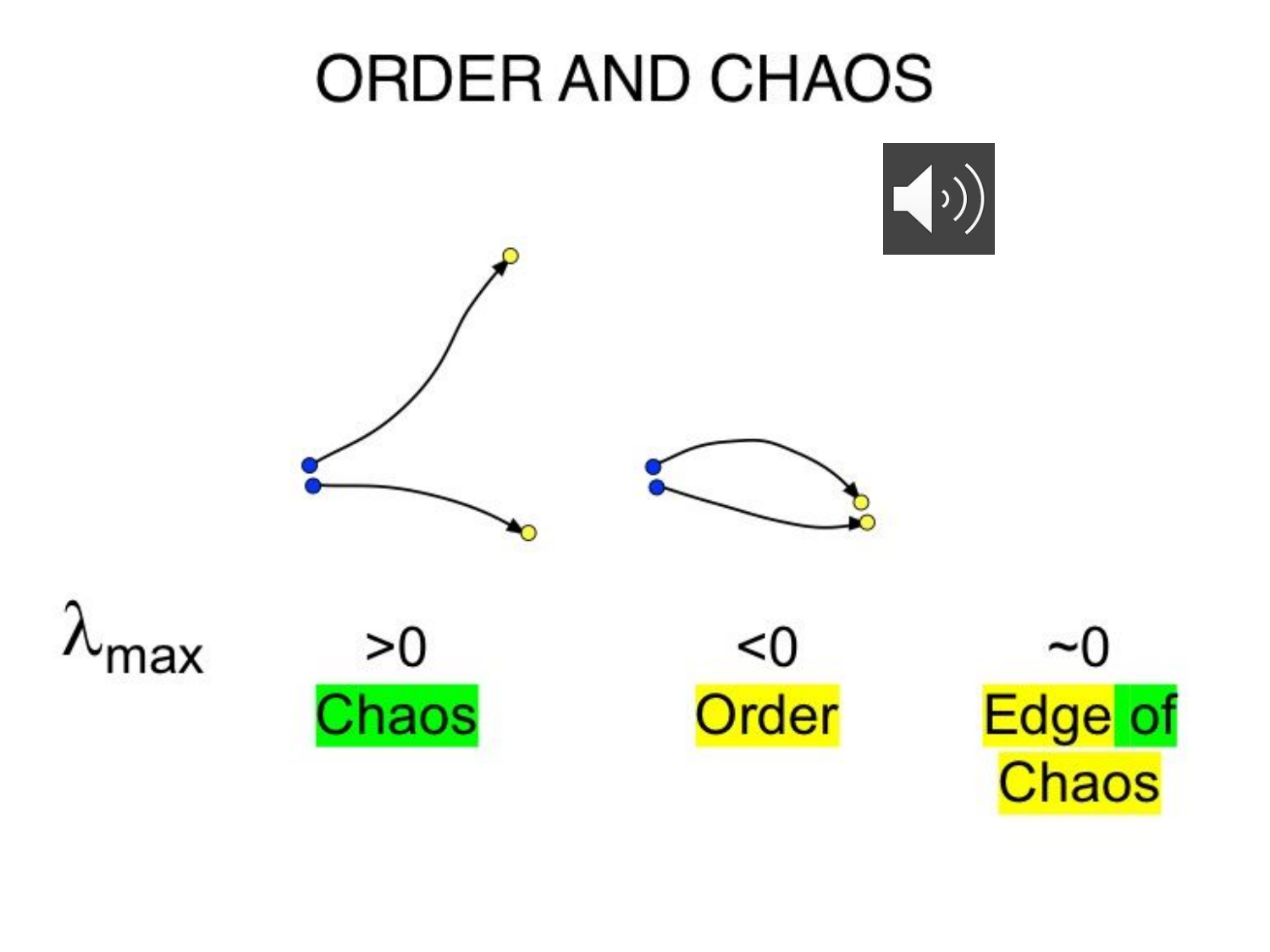

## Slide 31
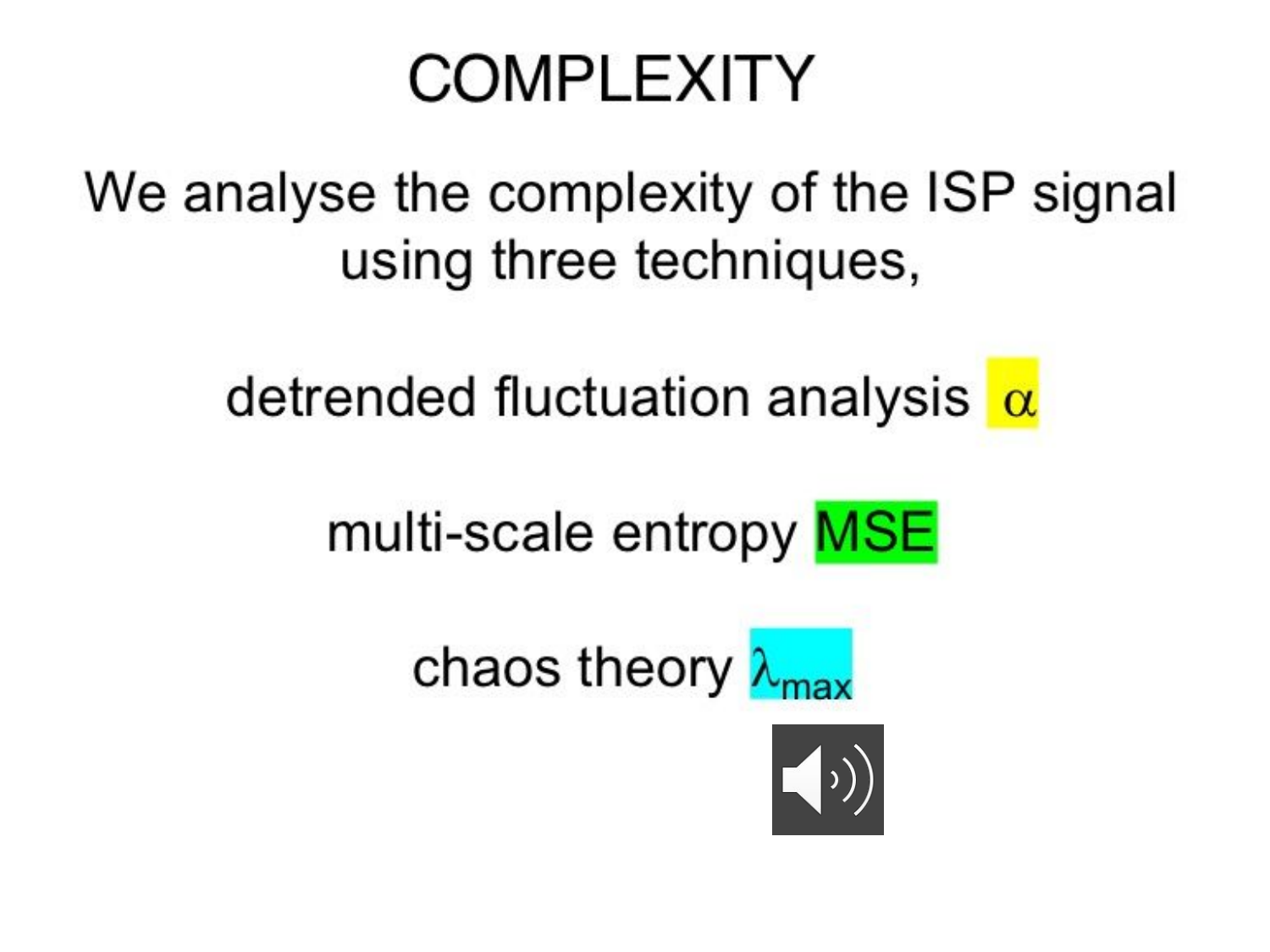

Supplement: Supplementary file 1 [file Presentation_1.PPTX]
